# Reproducibility and Robustness of a Liver Microphysiological System PhysioMimix LC12 under Varying Culture Conditions and Cell Type Combinations

**DOI:** 10.3390/bioengineering10101195

**Published:** 2023-10-14

**Authors:** Alicia Y. Lim, Yuki Kato, Courtney Sakolish, Alan Valdiviezo, Gang Han, Piyush Bajaj, Jason Stanko, Stephen S. Ferguson, Remi Villenave, Philip Hewitt, Rhiannon N. Hardwick, Ivan Rusyn

**Affiliations:** 1 Department of Veterinary Physiology and Pharmacology, Texas A&M University, College Station, TX 77843, USA; 2 Laboratory for Drug Discovery and Development, Shionogi Pharmaceutical Research Center, Shionogi & Co., Ltd., Osaka 561-0825, Japan; 3 Department of Epidemiology and Biostatistics, Texas A&M University, College Station, TX 77843, USA; 4 Global Investigative Toxicology, Preclinical Safety, Sanofi, Cambridge, MA 02141, USA; 5 Division of Translational Toxicology, National Institute of Environmental Health Sciences, Durham, NC 27709, USA; 6 Roche Pharma Research and Early Development, Roche Innovation Center Basel, F. Hoffmann-La Roche Ltd., 4070 Basel, Switzerland; 7 Chemical and Preclinical Safety, Merck Healthcare KGaA, 64293 Darmstadt, Germany; 8 Discovery Toxicology, Pharmaceutical Candidate Optimization, Bristol Myers Squibb, San Diego, CA 92121, USA

**Keywords:** microphysiological systems, liver, testing, tissue chip

## Abstract

The liver is one of the key organs for exogenous and endogenous metabolism and is often a target for drug- and chemical-driven toxicity. A wide range of experimental approaches has been established to model and characterize the mechanisms of drug- and chemical-induced hepatotoxicity. A number of microfluidics-enabled in vitro models of the liver have been developed, but the unclear translatability of these platforms has hindered their adoption by the pharmaceutical industry; to achieve wide use for drug and chemical safety evaluation, demonstration of reproducibility and robustness under various contexts of use is required. One of these commercially available platforms is the PhysioMimix LC12, a microfluidic device where cells are seeded into a 3D scaffold that is continuously perfused with recirculating cell culture media to mimic liver sinusoids. Previous studies demonstrated this model’s functionality and potential applicability to preclinical drug development. However, to gain confidence in PhysioMimix LC12’s robustness and reproducibility, supplementary characterization steps are needed, including the assessment of various human hepatocyte sources, contribution of non-parenchymal cells (NPCs), and comparison to other models. In this study, we performed replicate studies averaging 14 days with either primary human hepatocytes (PHHs) or induced pluripotent stem cell (iPSC)-derived hepatocytes, with and without NPCs. Albumin and urea secretion, lactate dehydrogenase, CYP3A4 activity, and metabolism were evaluated to assess basal function and metabolic capacity. Model performance was characterized by different cell combinations under intra- and inter-experimental replication and compared to multi-well plates and other liver platforms. PhysioMimix LC12 demonstrated the highest metabolic function with PHHs, with or without THP-1 or Kupffer cells, for up to 10–14 days. iPSC-derived hepatocytes and PHHs co-cultured with additional NPCs demonstrated sub-optimal performance. Power analyses based on replicate experiments and different contexts of use will inform future study designs due to the limited throughput and high cell demand. Overall, this study describes a workflow for independent testing of a complex microphysiological system for specific contexts of use, which may increase end-user adoption in drug development.

## 1. Introduction

The toxicological testing of drugs and chemicals using in vivo animal models is required for pre-clinical phases of drug development [1] and in chemical safety evaluations [2,3,4]. The challenges of translating in vivo animal data to human health decisions are well documented [5], and the legislative encouragement to reduce or eliminate animal testing is increasing in both the European Union [4,6] and the United States [7]. Consequently, the scientific community and regulatory bodies have embraced ambitious goals to develop so-called “new approach methods” (NAMs) for the efficacy and safety evaluation of chemicals and drugs [8,9,10,11,12].

NAMs span a wide range of in vitro, in chemico, and in silico methods. In vitro methods include both traditional static 2D cell cultures and more complex models, such as microtissues (e.g., spheroids and organoids) and microphysiological systems (MPS). MPS have been in active development over the past decade, stimulated by major investments from government agencies, private donors, and investors [13,14,15]. Many MPS, both academic and commercial, have been developed for a wide range of tissues [16], as well as two- [17] and multi-tissue models [18,19]. With the accelerated pace of MPS development, there has been an increase in interest in their qualification and use in regulatory and safety evaluations of chemicals and drugs [9,14,20]. However, a number of concerns regarding the implementation of MPS in safety and efficacy testing pipelines have been expressed by many end users [21,22], leading to a concerted effort by the manufacturers, end users, and regulators to inform the scientific community of how these models should be tested to demonstrate robustness, reproducibility, and utility for certain decision contexts [11,23,24,25]. Furthermore, several recent studies have provided clarity on the anticipated utility of liver MPS by investigating their performance with different cell sources [26,27,28]. These studies have also compared MPS to simpler culture methods, quantitatively assessing both the variability within and between studies. The aim of these investigations was to inform potential end users about the expectations, advantages, and challenges associated with MPS.

The liver is one of the organs where the development of alternative models capable of addressing contexts of use currently unmet by simpler models is of high interest [29], and many diverse liver MPS have been published [30]. The lack of consistency surrounding the characterization of the functionality of each of these models has been documented; suggestions on how these models should be tested before wide adoption have been offered by the end users [31]. Both academic publications reporting the development of new MPS and technical notes provided by MPS vendors accompanying their products offer some functional data and information on drug effects/metabolism data for liver MPS. However, there has been limited independent testing conducted to address the concerns regarding the robustness and reproducibility of these models. Additionally, there is a need to determine the most appropriate decision contexts and conditions of use for these MPS [20]. It is noteworthy that recent publications by the manufacturers of several liver MPS included data that shows performance across experiments and donors for several dozen compounds [32] or compared prediction accuracy for liver injury in different study designs (i.e., one replicate per drug vs. a dose–response) for over a hundred drugs [33]. Still, independent testing of several liver MPS to establish their functionality, reproducibility, robustness, and reliability not only adds useful information on what cell types and combinations may be most functional but also provides comprehensive data on the expected variability in these complex models and the challenges that end users may face [26,27,28].

One of the established perfused liver MPS models is the PhysioMimix LC12, a 12-well device comprised of primary hepatocytes, with or without nonparenchymal cells (NPCs), seeded into 3D perfusable scaffolds to form liver sinusoid-like structures, with circulating cell culture medium that continuously re-oxygenate the cells. The current technology, based on the LiverChip design developed by the Griffith lab over 10 years ago [34,35], has been used in a number of toxicological and toxicokinetic studies [36,37,38,39,40]. The current commercial version of this MPS, PhysioMimix LC12, has been collaboratively tested by the manufacturer and the US Food and Drug Administration [41] and also independently at the Roche Innovation Center Basel [42]. While previous studies provide robust and diverse data to assess the suitability of this model for drug pharmacokinetics and hepatotoxicity studies, each published report included a limited number of replicate experiments. As a result, quantitation of inter- and intra-experimental variability is difficult. Moreover, these studies solely utilized primary human hepatocytes (PHHs) with minimal or no inclusion of physiologically proportional levels of non-parenchymal cells (e.g., Kupffer cells) and did not typically include comparisons with other liver MPS models or conventional 2D systems. 

To add to the previous body of literature regarding the performance of liver MPS and to provide critical new data on the robustness and reproducibility of the PhysioMimix LC12 for toxicological studies of the liver, we conducted multiple replicate studies lasting an average of 14 days in culture. These studies used either PHHs or human induced pluripotent stem cell (iPSC)-derived hepatocytes, with and without combinations of NPCs. We evaluated both basal function and metabolic capacity and characterized the model performance with various cell combinations, under intra- and inter-experimental replication, and compared them to multi-well plates and other MPS. Additionally, we conducted power analysis to determine the minimum number of independent replicates required to show statistical significance for changes in specific biomarkers related to hepatic function, drug metabolism, and immune-mediated hepatotoxicity.

## 2. Materials and Methods

### 2.1. Overall Experimental Design

**Figure 1:**
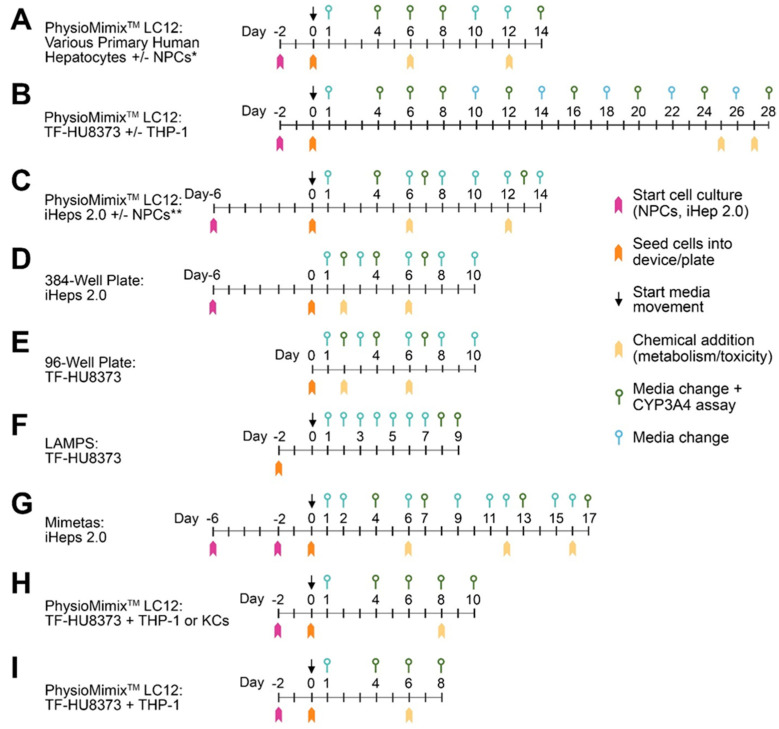
General study designs for the experiments performed. The range of experimental conditions that was utilized in this study to evaluate PhysioMimix LC-12 and other liver in vitro models. See detailed experimental protocols for each experiment detailed in Supplemental Table S1. Day 0 corresponded to the day when the cells were seeded into devices and media movement commenced (for microfluidic models) or cells were seeded into multi-well plates (for static cultures). In the PhysioMimix LC-12, media flow through the device/scaffolds without cells was initiated before cells were seeded, according to the manufacturer’s protocols. Media changes with or without CYP3A4 assay and the timing of drug/chemical additions are indicated (see symbols legend in the inset). (**A**) The most typical PhysioMimix LC-12 study design spanning a total of 14 days in culture. In the experiments that used NPCs, they were started in culture 2 days prior to the seeding of all cells into the device and initiation of flow. The asterisks (*, **) denote that different types of NPCs were used for PHHs and iHeps 2.0 (see Section 2.1). (**B**) The PhysioMimix LC-12 study extended to 28 days. (**C**) Studies with iHeps 2.0 in the PhysioMimix LC-12 model. (**D**) Static culture in 384-well plates for iHeps 2.0. (**E**) Static cultures in 96-well plates for PHHs (HU8373 donor only). (**F**) Monoculture PHH (HU8373) study in a LAMPS model. (**G**) Study with iHeps 2.0 in the OrganoPlate 2-lane 96 (Mimetas, Leiden, The Netherlands) model. (**H**) Study of Trovafloxacin +/− LPS in PhysioMimix LC-12 model. (**I**) Study of the metabolism of a pesticide mixture in PhysioMimix LC-12 model.

This study consisted of multiple rounds of experiments that probed the performance of different lots of PHHs and iPSC-derived hepatocytes, cell combinations, and toxicological contexts of use. Various combinations of cell types were seeded into the PhysioMimix LC12 and cultured for a duration of 8–28 days. Typical experimental designs for different contexts of use are shown in Figure 1. Exact experimental designs, detailed study protocols, and all experimental data are available (see Supplemental Table S1 for hyperlinks). After cells were seeded, media changes were performed every 1 to 3 days, with a collection of effluent for biochemical assays to assess biomarkers of liver cell function. The latter was examined using albumin and urea secretion as markers of hepatic function and LDH activity as a marker of cell viability. CYP3A4 activity was measured using a live, luminescent assay with Luciferin IPA as a marker for metabolic capacity.

These experiments examined various utilization scenarios, including the exploration of diverse combinations of cell types (Supplemental Table S2). The testing of various combinations of cell types was used to determine if immortalized cell lines may be sufficient as NPCs and to assess potential differences in hepatic function between monocultured and co-cultured PHHs and iPSC-derived hepatocytes within the PhysioMimix LC12. Two main types of human liver parenchymal cells were used: PHHs (from 5 different donors) and iPSC-derived hepatocytes. Both monoculture and co-culture (with NPCs) conditions were tested. For co-culture with PHHs, either (i) THP-1 cells differentiated with phorbol 12-myristate 13-acetate into macrophage-like cells (referred to herein as THP-1 cells); (ii) Kupffer cells; (iii) Kupffer cells and hepatic stellate cells (HSC); or (iv) Kupffer cells, HSC, and liver sinusoidal endothelial cells (LSEC) were seeded. For the co-culture of iPSC-derived hepatocytes, cells were co-seeded with THP-1 and HMEC-1 cells. 

Metabolic capacity was assessed across the various cell type conditions, including both types of hepatocytes. Midazolam (MDZ) was tested as a representative substrate for CYP3A4 [43]. Cells were treated with MDZ for up to 24 h on different days of culture, with sampling at 2, 6, and 24 h after treatment. The collected media was processed using high-performance liquid chromatography tandem mass spectrometry (HPLC-MS/MS) to determine the concentration of both the remaining parent compound (MDZ) and metabolites formed (1′-OH MDZ and 1′-OH MDZ glucuronide). We also tested the metabolism of equimolar mixtures (1 or 5 µM) of 20 pesticides that were previously used to evaluate the metabolic competence of different in vitro liver models [44]. Cells were treated on day 6 of culture, and media were collected for analysis 48 h thereafter. Samples were analyzed using either gas chromatography (GC)-MS/MS or HPLC-MS/MS to determine whether parent molecule concentrations diminished, consistent with the metabolic activity of the cells.

For each optimized cell type combination, the variability and reproducibility of the functional and metabolic parameters were evaluated via repeated studies. Both intra- and inter-experimental variability was evaluated across time in culture. The coefficient of variation was calculated and compared to the benchmark of 30% or less proposed for liver MPS [31]. Power analyses were performed to determine the minimum number of replicates required to achieve statistical differences in hepatic function and metabolism over time.

### 2.2. Chemicals and Materials

Midazolam (19391, CAS#59467-70-8) and 1-hydroxymidazolam (10385, CAS#59468-90-5) were purchased from Cayman Chemical (Ann Arbor, MI, USA); 1-hydroxymidazolam beta-D-glucuronide (CAS#81256-81-7) was kindly provided by Sanofi (Paris, France). Lipopolysaccharide (LPS, Cat#L2630) and Trovafloxacin mesylate (Cat# PZ0015) were purchased from Millipore Sigma (St. Louis, MO, USA). The twenty pesticides that were used in this study as analytes or standards (Supplemental Table S3) were purchased from Millipore Sigma, or Chem Service (West Chester, PA, USA). Analytical chemistry reagents methanol (Cat No.: 646377), acetonitrile (Cat No.: 34998), pentane (Cat No.: 34956), diethyl ether (Cat No.: 309966), and distilled water with 0.1% formic acid (Cat No.: 576913) were purchased from Millipore Sigma.

The microfluidic tissue chips used in this project were purchased from either CNBio (PhysioMimix LC12, Cambridge, UK), Mimetas (OrganoPlate^®^ 2-lane 96, Leiden, The Netherlands), or Nortis Bio (SCC-001, Bothell, WA, USA). A PhysioMimix LC12 plate has the footprint of a traditional 96- or 384-well plate and contains 12 individual devices. Each chip contains a media reservoir and a culture well consisting of a plastic retaining ring that holds a scaffold and filter in place. Cells were seeded onto and cultured within the scaffold. The plate was attached to a pneumatic docking station, which has micropumps that produce recirculating media flow, which is adjustable using the PhysioMimix LC12 controller. The OrganoPlate^®^ 2-lane 96 plate contains 96 devices, each of which contains one gel channel and one perfusion channel [33] to enable the culture of a perfused tubule adjacent to the extracellular matrix without a membrane. The “liver acinus MPS” (LAMPS) model was based on the Nortis SCC-001 device that contains a central growth area where cells and extracellular matrix are sequentially layered [45]. Black-walled, clear-bottom, tissue culture-treated 96-well plates (3603, Corning, Corning, NY, USA) and 384-well plates (3764, Corning) were used for 2D cell culture experiments.

### 2.3. Cells and Cell Culture Reagents 

All cells used in these studies are listed in Supplemental Table S2. Primary human hepatocytes were obtained from ThermoFisher (HMCPIS, Lots #HU8373 and #HU8300, Waltham, MA, USA), Lonza (HUCPG, Lots #HUM183231 and #HUM182531, Basel, Switzerland), and LifeNet Health (Lot #212782, Virginia Beach, VA, USA). Primary human hepatocyte plating media consisted of William’s E media (A1217601, ThermoFisher) supplemented with thawing and plating supplements (3.6% cocktail A, 5% FBS, 10 nM dexamethasone (CM3000, ThermoFisher) and was used to plate and seed cells. Primary human hepatocyte maintenance media consisted of William’s E media with primary hepatocyte maintenance supplements (4% cocktail B, 1 nM dexamethasone; CM4000, ThermoFisher) and was used for cell culture from day 1 until the end of each experiment. 

Human iPSC-derived hepatocytes were purchased from FujiFilm-Cellular Dynamics International (C1023, iCell Hepatocytes 2.0, Lot# 103934, Santa Ana, CA, USA). iPSC-derived hepatocyte plating media consisted of RPMI 1640 (11835030, ThermoFisher) supplemented with 2% B-27 supplement (17504044, ThermoFisher), 2% iCell Hepatocytes 2.0 Medium Supplement (M1024, FujiFilm-Cellular Dynamics International), 100 nM dexamethasone (265005, Millipore Sigma), 25 µg/mL gentamicin (15710072, ThermoFisher), and 20 ng/mL oncostatin M (295-OM-010, R&D Systems, Minneapolis, MN, USA), and was used for pre-differentiation and plating of cells according to the manufacturer’s protocol. iPSC-derived hepatocyte maintenance media consisted of RPMI 1640, 2% B-27, 2% iCell Hepatocytes 2.0 Medium Supplement, 100 nM dexamethasone, and 25 μg/mL gentamicin, and was used for cell culture from day 1 until the end of each experiment. Primary human Kupffer cells (NPC-AD-KC, Lot #2118082) and primary human stellate cells (HSC, NPC-AD-SC-P0, Lot #2118082p0) were obtained from LifeNet Health (Virginia Beach, VA). Primary human liver sinusoidal endothelial cells (LSEC, ACBRI 566, Lot #566.01.01.01.1T) were obtained from Cell Systems (Kirkland, WA, USA). Kupffer cells were cultured in Advanced DMEM (12491015, ThermoFisher), 5% fetal bovine serum, and 3.6% Cocktail A (CM3000, ThermoFisher). HSCs were cultured in DMEM (11965092, ThermoFisher), 10% fetal bovine serum, and 1% antibiotic/antimycotic cocktail (15240062, ThermoFisher). LSEC were cultured in Complete Classic Medium with Serum and CultureBoost™ (4Z0-500, Cell Systems) with 1% CultureBoost, and 0.2% Bac-Off^®^ supplements (4Z0-643, Cell Systems). HSC and LSEC purity were verified to be >95% for both cell types (data not shown) using fluorescence-activated cell sorting using anti-human α-SMA (NBP2-34522AF488, Novus Biologicals, Centennial, CO, USA) and CD-31 (ab215912, Abcam, Cambridge, UK) primary antibodies. Samples were analyzed using a Cytek Amnis CellStream Flow Cytometer (Cytek Biosciences, Fremont, CA, USA), and excitation lasers (488 and 647 nm) were used to image single cells. A-SMA (488 nm) and CD-31 (642 nm) channels were used for data collection. Color compensation was not necessary as no overlapping emission spectra were observed.

THP-1 monocytes and HMEC-1 endothelial cells were obtained from ATCC (Manassas, VA, USA). THP-1 monocytes were cultured in RPMI (30-2001, ATCC) with 10% FBS (30-2020, ATCC), 1% Pen-strep (15140148, ThermoFisher), and 2 mM L-glutamine (25030149, ThermoFisher). THP-1 monocytes were differentiated [45] into mature, adherent macrophages by treatment with 200 ng/mL phorbol 12-myristate-13-acetate (356150050, ThermoFisher) for 48 h before seeding. HMEC-1s were cultured in MCDB 131 medium (10372019, ThermoFisher) with 10% FBS, 2 mM L-glutamine, 100 units/mL penicillin-streptomycin, 1 µg/mL hydrocortisone (H0888, Millipore Sigma), and 10 ng/mL epidermal growth factor recombinant human protein (PHG0314, ThermoFisher). 

### 2.4. PHH and NPC Culture Methods in the PhysioMimix LC12 and 96-Well Plates

The day when PHHs were seeded into PhysioMimix LC12 plates was defined as day 0 (Figure 1). PHHs were cultured in the PhysioMimix LC12 following the manufacturer’s protocols. PHHs were thawed into CHRM (CM7000, Thermo Fisher), centrifuged at 100× *g* for 10 min, resuspended in plating media, and then seeded into the PhysioMimix LC12 scaffold at a seeding density of 475,000–600,000 cells/chip. The flow was initiated at 1 µL/s. 

For co-culture conditions, THP-1 monocytes and HMEC-1 endothelial cells were seeded either on day 0 or 1 at a seeding density of 60,000–160,000 cells/chip or 60,000 cells/chip, respectively. Primary KCs, HSCs, and LSECs were seeded either on day 0 or 1 at a seeding density of 100,000 cells/chip, 35,000 cells/chip, and 70,000–140,000 cells/chip, respectively. Cell seeding ratios were determined based on previous experiments [26,27] or reports of the physiological cell ratios in the human liver [30]. Media were collected and exchanged every 1–3 days by aspirating and replacing media from the media reservoir (1.8 mL/chip). 

For the PHH 2D model, 96-well plates were first coated with 200 μg/mL collagen I (CB354249, Corning Inc.) and 50 μg/mL fibronectin (F1141, Sigma-Aldrich, St. Louis, MO, USA) and incubated at 37 °C, 5% CO_2_ for 1 h. Thawed PHHs were collected by centrifugation (100× *g*, 10 min), resuspended in plating media, and seeded at a density of 50,000–60,000 cells/well. After incubation overnight, the media were changed to maintenance media. The total well volume was 100 μL, and plates were incubated at 37 °C, 5% CO_2,_ with media refreshed every 1–2 days.

For evaluation of MDZ metabolism, cells were exposed on days 4–16 of culture to 5 µM MDZ (dissolved in methanol for the final concentration of 0.16% *v*/*v*). After drug addition, media were collected every 2, 6, and 24 h. After 24 h, the drug treatment media were replaced with fresh maintenance media. For the experiment with pesticide mixtures, we selected chemicals from the Agency for Toxic Substances and Disease Registry Substance Priority List, which contains compounds that are commonly detected at Superfund sites and are known to be hazardous to human health [46]. A molar-equivalent mixture was created by combining all 20 pesticides and diluting them to a final concentration of 1 or 5 μM each in dimethyl sulfoxide (DMSO, final concentration 0.5% *v*/*v*). Media was collected 48 h after treatment for the analyses detailed below. For evaluation of immune-mediated toxicity with Trovafloxacin and LPS treatment, PHHs + THP-1 monocytes or PHHs + KCs were exposed on day 6 of culture to 25 or 100 µM of Trovafloxacin, with or without LPS. After 48 h, the drug treatment media were collected for biochemical assays detailed below.

### 2.5. iPSC-Derived Hepatocytes and NPC Culture Methods in the PhysioMimix LC12 and 96-Well Plates

Before seeding into the PhysioMimix LC12 platform, iPSC-derived hepatocytes were pre-differentiated according to the manufacturer’s protocols as follows. iPSC-derived hepatocytes were thawed, cells counted (average viability 85.1 ± 5.5%), and seeded at a density of 2.5 × 10^6^ viable cells/well on a 6-well plate pre-coated with type 1 collagen (657950-005, Greiner Bio-One North America, Monroe, NC, USA) in plating media and cultured for 4 h, after which unattached cells were removed with a media change. iPSC-derived hepatocyte cells were pre-differentiated for 5 days with daily changes in plating media [26]. The differentiated clusters were collected by centrifugation (200× *g*, 3 min) and resuspended in maintenance media, and then seeded into the PhysioMimix LC12 scaffold at a seeding density of 900,000 cells/chip. The day when the iPSC-derived hepatocytes were seeded into PhysioMimix LC12 plates was defined as day 0 (Figure 1). After incubation for 1 h, flow was initiated (1 µL/s). For co-culture conditions, THP-1 monocytes and HMEC-1 endothelial cells were seeded either on day 0 or 1 at a seeding density of 90,000 cells/chip based on previous studies [33]. Media were collected and exchanged every 1–3 days by aspirating and replacing media from the media reservoir (1.8 mL/chip). 

For the iPSC-derived hepatocytes 2D culture experiments, a 384-well plate was coated with 200 μg/mL collagen I (CB354249, Corning Inc.) and 50 μg/mL fibronectin (F1141, Sigma-Aldrich, St. Louis, MO, USA) and incubated at 37 °C, 5% CO_2_ for 1 h. Differentiated clusters were collected as previously described, resuspended in maintenance media, and seeded at a density of 100,000 cells/well. The total well volume was 100 μL, and plates were incubated at 37 °C, 5% CO_2,_ with media refreshed every 1–2 days.

### 2.6. iPSC-Derived Hepatocytes Culture Methods in the OrganoPlate^®^ 2-Lane 96

iPSC-derived hepatocytes were cultured in the OrganoPlate^®^ 2-lane 96 (Mimetas, Leiden, The Netherlands) using a previously established protocol [26]. Day 0 was defined when cells were seeded into OrganoPlate^®^ 2-lane 96 plates. Differentiated clusters were collected as previously described and resuspended into 3.33 mg/mL collagen (Cultrex 3-D Culture Matrix Rat Collagen-I, 3447-020-01, R&D Systems; 5 mg/mL type 1 collagen, 1 M HEPES, 37 g/L sodium bicarbonate at a ratio of 4:1:1, respectively) at a density of approximately 8.0 × 10^6^ cells/mL. The iPSC-derived hepatocyte/collagen suspension (2.5 μL/device) was injected into the inlet of the gel channel in each of the 96 devices on the plate using an electronic pipettor. The plate was then incubated at 37 °C, 5% CO_2,_ for 15 min to allow for collagen polymerization. For co-culture conditions, a mixture of HMEC-1 endothelial cells at 40 × 10^6^ cells/mL and THP-1 monocytes at 3 × 10^6^ cells/mL was prepared in iPSC-derived hepatocyte maintenance media, and 2.5 μL was injected into the inlets of the perfusion channel of each device using an electronic pipettor. The plates were incubated at a 70° angle at 37 °C, 5% CO_2_ to allow attachment of the iPSC-derived hepatocytes in the gel channel. After a 15 min incubation, 50 µL of iPSC-derived hepatocyte maintenance media was added into medium inlets and outlets of the perfusion channel, and the plates were incubated at a 70° angle at 37 °C, 5% CO_2_ for an additional 45 min. The plates were then placed on the perfusion rocker platform (Mimetas) set to cycle every 4 min to a maximum angle of approximately 15° to induce gravity-driven media to flow through the perfusion channel. The media was collected and exchanged every 1–2 days (Figure 1) by aspirating and replacing media from medium inlets and outlets (50 µL each).

### 2.7. PHH Culture Methods in a Human Microfluidic four-Cell LAMPS Model

PHHs were cultured in microfluidic tissue chips (SCC-001, Nortis Bio) using a previously established protocol [27]. Device chambers were coated with a mixed solution of 50 μg/mL fibronectin and 200 μg/mL collagen I in PBS and incubated for 1 h at room temperature. Once the devices were coated, PHH (TU-HU8373) was injected at a density of 2.75 × 10^6^ cells/mL (150 μL/chip) in PHH plating media (see above) and incubated overnight (37 °C, 5% CO_2_). The following morning, a 2.5 mg/mL solution of pH 7.2 rat tail collagen/10 mM HEPES/HBSS was injected into the devices (150 μL/chip). Chips were then inverted and incubated for 1 h at 37 °C. After this short incubation, chips were re-inverted and incubated overnight at 37 °C.

The next morning, device tubing was prepared as follows: inlet tubing was prepared by cutting 10-inch sections of polyetheretherketone (PEEK) tubing (1569XL, IDEX, Lake Forest, IL, USA), and fitting 1 inch of c-flex tubing (06422–00, Cole-Parmer, Vernon Hills, IL, USA) to each end with a catheter blunt (SC 20/15, Instech, Plymouth Meeting, PA, USA) on one end to interface with the inlet of the chip (the other c-flex section would fit over a needle blunt on the media syringe). Outlet tubing was prepared by cutting 2-inch sections of PEEK tubing and fitting a 1-inch section of c-flex tubing to one end with a catheter blunt to interface with the outlet of the chip. The other end of the PEEK tubing was placed into a glass vial with a pre-slit cap. All tubing was autoclaved and primed with cell culture media prior to chip connection. PHH maintenance media was perfused using syringe pumps (Fusion 200, Chemyx, Stafford, TX, USA) at a rate of 15 μL/h for 9 days. Each day, 360 μL of effluent was collected for analysis.

### 2.8. Biochemical Assays

Cell culture media was collected every time media was exchanged (Figure 1) and stored frozen at −80 °C until analyses for various biomarkers, including albumin, urea, lactate dehydrogenase (LDH), and human interleukin-6 (IL-6). The ELISA assays for albumin (E88-129, Bethyl Laboratories, Montgomery, TX, USA), urea (EIABUN, ThermoFisher), LDH (ab102526, Abcam, Cambridge, UK), and IL-6 (D6050, R&D Systems) were conducted using manufacturer’s instructions. For CYP3A4 activity, the P450 Glo 3A4 with Luciferin-IPA assay (V9001, Promega, Madison, WI, USA) was used; this is a live cell assay, and it was conducted according to the manufacturer’s instructions as follows. The Luciferin-IPA substrate was diluted 1:1000 in maintenance media and added to chips or wells 1 h prior to reading luminescence using a plate reader (SpectraMax iD3, Molecular Devices, San Jose, CA, USA). After the assay, the media was replaced. 

### 2.9. Immunocytochemical Staining

At the conclusion of the cell culture period, scaffolds were removed from the PhysioMimix LC12 plate and placed in a 24-well plate containing phosphate-buffered saline (PBS). After washing with PBS twice, scaffolds were fixed by adding 4% formalin solution for 10 min. Scaffolds were washed again with PBS to remove the formalin and treated for 10 min with 0.1% Triton X-100 (BP151, ThermoFisher) in PBS to permeabilize cells. Cells were washed again with PBS and incubated for 60 min in 2% bovine serum albumin (A8806, Millipore-Sigma) in PBS blocking solution. An anti-CYP3A4 polyclonal antibody (18227-1-AP, ThermoFisher), anti-ZO-1 monoclonal antibody (ZO1-1A12, ThermoFisher), anti-Human Albumin antibody (CL2513A, Cedarlane, Burlington, NC, USA), anti-ABCB11/BSEP antibody (ab155421, Abcam), and anti-MRP2 antibody (MAB4150, Sigma Aldrich) were added to label CYP3A4 metabolic enzymes, tight junctions, albumin proteins, and transporter proteins, respectively. Plates were incubated overnight at 4 °C and goat anti-Mouse IgG secondary antibody (ab150115, AF647, Abcam) and goat anti-Rabbit IgG secondary antibody (ab150079, AF647, Abcam) were added to visualize CYP3A4, ZO-1, BSEP, Albumin, and MRP2. Plates were incubated for 1.5 h at room temperature. All scaffolds were counterstained with FITC-phalloidin (F432, ThermoFisher) to visualize the cytoskeleton and incubated for 0.5 h at room temperature. Scaffolds were then mounted on a glass slide using ProLong Gold Antifade mountant with DAPI (P36935, ThermoFisher), which counterstained cells with DAPI to visualize nuclei. Cells were imaged using the ImageXpress Micro (Molecular Devices). 

### 2.10. Sample Preparation for the Analytical Chemistry Analyses

After treatment with MDZ or pesticide mixtures (Supplemental Table S3), 50 µL media samples were mixed with 100 µL of 0.1 or 1 µM internal standard (Caffeine-13C3, C-082, Millipore Sigma; Supplemental Table S3) solution in acetonitrile on ice, respectively. Samples were vortexed and centrifuged (12,000× *g*, 10 min, room temperature) to allow for protein precipitation. The supernatant was collected from each sample, dried under vacuum, and reconstituted in 50 µL aqueous mobile phase. The reconstituted samples were transferred to glass autosampler vials with 200 µL inserts and stored at −20 °C prior to HPLC-MS/MS analysis. After treatment with pesticide mixtures, 50 μL media samples were spiked with 10 μL of 10 μM internal standards, mixed with 50 μL of methanol and 200 μL of pentane: diethyl ether (1:1, *v*/*v*), vortexed, and then centrifuged at 600× *g* for 5 min. Supernatants were transferred to an amber vial for GC-MS/MS analysis.

### 2.11. Determination of Midazolam and Metabolites and Pesticide Metabolism Using HPLC-MS/MS

For the determination of MDZ and its metabolites using HPLC-MS/MS, 8 µL of each sample was automatically injected and chromatographed on a ZORBAX SSHD Eclipse Plus C18 column (3.0 × 50 mm, 1.8 µm, 959757-302, Agilent, Santa Clara, CA, USA) using a 1290 Infinity II LC (Agilent) with a guard column (2.1 × 5 mm, 1.8 µm, 821725-901, Agilent). Column temperature was set at 40 °C and flow rate to 0.4 mL/min. Chromatographic conditions started at 90% mobile phase A (water with 0.1% (*v*/*v*) formic acid) and 10% mobile phase B (acetonitrile with 0.1% (*v*/*v*) formic acid) for 1 min, increased to 80% B by 3 min, to 95% B by 4 min, and then returned to and stayed at initial conditions at 5 to 8 min for equilibration prior to the next run. MS/MS analyses were performed using an Agilent 6470 triple quadrupole MS (Agilent) in positive ion mode with an electrospray ionization source. Capillary voltage, sheath gas pressure, and sheath gas temperature were set at 3500 V, 40 psi, and 350 °C, respectively.

For the determination of pesticide metabolism, chromatographic conditions were as previously reported in [47], and HPLC-MS/MS analysis was performed using the instrument and columns mentioned above. Analytical response was acquired in positive or negative ion modes. Chromatographic conditions started at 98% mobile phase A (water with 0.1% (*v*/*v*) formic acid) and 2% mobile phase B (methanol with 0.1% (*v*/*v*) formic acid) for 1 min, increased to 80% B by 3 min, to 95% B by 4 min, and then returned to initial conditions at 5 min and held for an additional 3 min for a total run time of 8 min per sample at a flow rate of 0.4 mL/min. For negative ion compounds, the LC gradient and flow rate were the same as in positive mode, except that mobile phase A was water and mobile phase B was acetonitrile.

### 2.12. Determination of Pesticide Metabolism GC-MS/MS

Detection of analytes was achieved using a 7890B GC and 7010B GC/MS triple quadrupole mass spectrometer (Agilent Technologies, Santa Clara, CA, USA), as detailed in [47]. Samples (1 μL) were injected in splitless mode, and the analytes were separated with a VF-5 ms GC column (60 m × 250 μm × 0.25 μm, Agilent Technologies) and ionized with an electron ionization source. The column head pressure was set at 21.5 psi with a constant flow rate of 1.2 mL/min using helium gas. Initial column temperature was held at 70 °C for 5 min, increased to 150 °C at 50 °C/min, ramped to 280 °C at 4 °C/min, and then held for 15 min. The total run time was 42.1 min. The injector temperature was set at 250 °C. The ion source and auxiliary transfer line temperatures were 300 °C. Electron multiplier voltage was set at 1884 V. Nitrogen gas was used as the collision gas for all MS/MS experiments, and the pressure of collision gas was set at 16.8 psi.

### 2.13. Statistical Analyses 

General descriptive statistical analyses were conducted using GraphPad Prism 9.2 (San Diego, CA, USA). Statistical significance (*p* < 0.05) was tested with one-way ANOVA with Dunnett’s multiple comparisons test, as indicated in Figure legends. Multi-factor analysis of variance was used to decompose variation in the measurement from different days, experiments, and other factors. Power analyses for sample size needed to reach statistical significance with 80% statistical power were estimated using (i) the two-sided paired *t*-tests and (ii) two-sided two-sample *t*-tests with different variances for paired and independent samples, respectively. MATLAB (v. R2018a), SAS (v. 9.4), and PASS (v. 15) software packages were used to estimate the effect size and calculate the required sample size [48,49]. 

## 3. Results

This study investigated several decision contexts and conditions of use for the PhysioMimix LC12 model as an in vitro platform for studies of liver function, chemical metabolism, and drug effects (Figure 1). With respect to the decision contexts, we investigated (i) whether the model is robust and reproducible in terms of both synthetic and metabolic liver function when cells from different donors are used, (ii) the physiological and metabolic function of this model over time, (iii) the utility of this model in studies of drug metabolism and pharmacokinetics with different classes of compounds, and (iv) whether this model can replicate innate immune-mediated liver toxicity. With respect to the conditions of use, we (i) explored different types of cells, including both primary and iPSC-derived hepatocytes, along with various combinations of NPCs and (ii) conducted comparisons between the PhysioMimix LC12 and other liver MPS models, as well as traditional multi-well plate cultures. 

### 3.1. Comparison of Cell Morphology and Seeding Density by Cell Source

**Figure 2:**
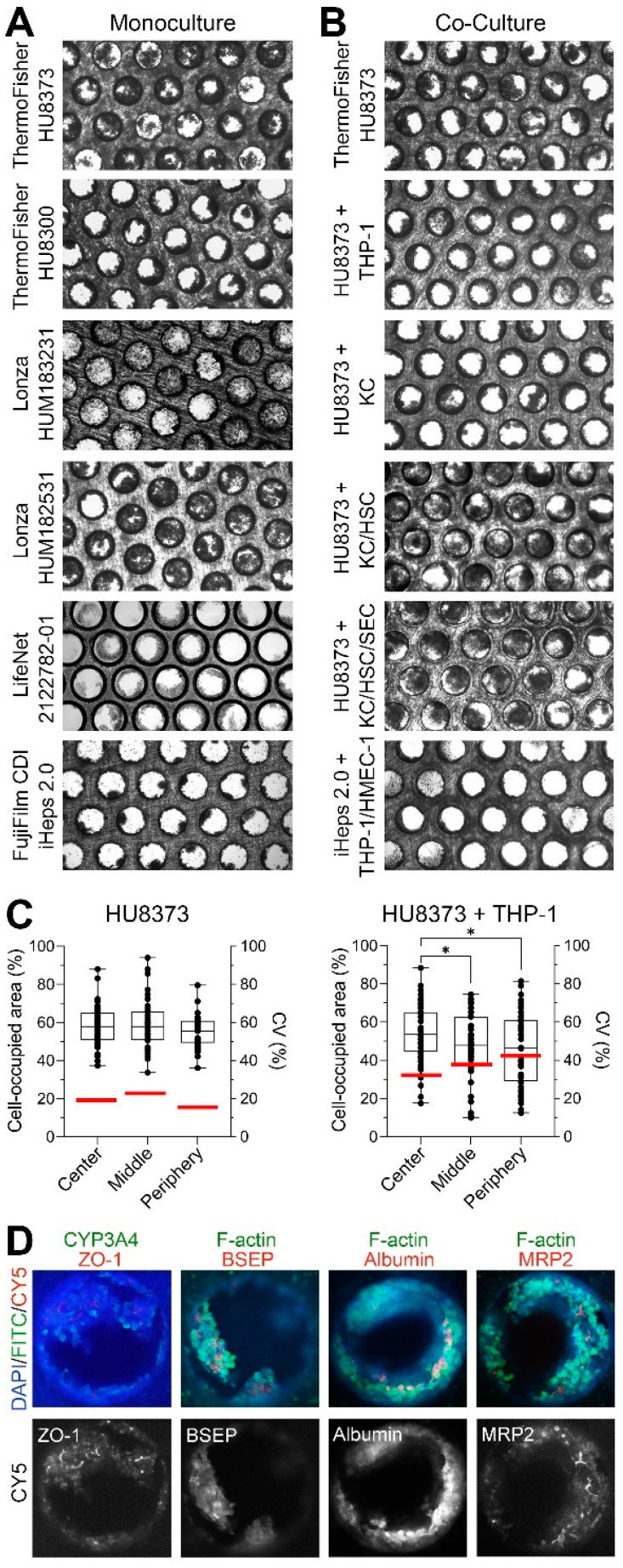
Comparison of morphology and cell seeding density in the PhysioMimix LC-12 model. (**A**,**B**) Bright field images of the representative PhysioMimix LC12 scaffolds removed from plate on day 14 of culture (See Figure 1A,C) and representing different cell types. Shown are the monocultures (**A**) with different primary PHHs and iPSC-derived hepatocytes (iHeps 2.0). Co-culture of HU8373 PHHs with different combinations of NPCs, and iHeps 2.0 with THP-1/HMEC-1 cells are shown in (**B**). (**C**) Quantitative analysis (left y-axis) of the cell-occupied areas in the sub-regions of the representative scaffolds on day 14 in culture with HU8373 PHHs cultured alone (**left**) or with THP-1 cells (**right**). Box-and-whisker plots show the median (line), inter-quartile range (box), and range (whiskers) with individual channel values shown as dots. Asterisks (*) denote statistical differences between sub-regions (*p* < 0.05, one-way ANOVA with Dunnett’s multiple comparisons). Right y-axes show the coefficient of variability for each sub-region (thick red horizontal line). (**D**) Immunofluorescent staining for cell nuclei (DAPI, blue), CYP3A4 or F-actin (FITC, green), and ZO-1, BSEP, Albumin or MRP2 (CY5, red) in representative channels seeded with PHH (HU8373) after 14 days of culture. Top row shows a composite image of all stains. Bottom row shows CY5 channel.

A unique feature of both the LiverChip and PhysioMimix LC12 versions of this liver MPS is the micro-channeled scaffold through which media is constantly re-circulated. Tissue structures that are formed in these microchannels, ~300 in each scaffold, have been characterized using optical and electron microscopy [36] and are routinely used to assess seeding quality [38,39,42,50] or explain the potentially poor performance of the individual scaffolds in each 12-scaffold device [41]. In our study, several PHH donors (selected based on vendor-provided characterization data relating to plating efficiency, high cell viability after thawing (>80%) and verified for induction of major CYPs), as well as iPSC-derived hepatocytes (iHeps 2.0) were tested in combination with different NPCs; therefore, we first evaluated tissue morphology. Representative scaffold images are shown in Figure 2A,B to demonstrate the differences in tissue micro-structures among donors and cell combinations. Cells from all five tested PHH donors formed different structures with the perfused micro-channels of various diameters and shapes, likely impacting media accessibility to the cells. While combinations of PHH and one NPC cell type (THP-1 or Kupffer cells) were largely indistinguishable from PHH-monoculture conditions, the addition of HSCs with or without LSECs resulted in obstruction of the microchannels. iHeps 2.0 seeded as a monoculture after differentiation (Figure 1) remained as cell aggregates and did not form a continuous coating of the channels. The addition of THP-1 and HMEC-1 cells with iHeps 2.0 resulted in some improvement to the contiguous cell lining of the microchannels. 

Due to the manual method of cell seeding in this device (slowly pipetting cells onto the scaffold in either a “star” or “8” shape to cover the maximum scaffold area), we observed not only heterogeneity in terms of seeding density and micro-tissue morphology between scaffolds but also among the microchannels in each scaffold. To evaluate cell seeding density and distribution in the individual scaffolds, we estimated the cell-occupied area for each of the 300 microchannels (Figure 2C). This was investigated for PHH monoculture (donor HU8373) and co-culture (HU8373 + THP-1 cells) conditions. Microchannels were assigned to 3 concentric areas in the scaffold with approximately equal numbers of microchannels: the center, middle, and periphery. For PHH monocultures, the median cell-occupied area (50–60%) was consistent across the center, middle, and periphery of the scaffold; however, the values varied widely, with an average coefficient of variability (CV) of around 20%. For PHH + THP-1 cultures, significant differences in the mean cell-occupied area were observed, diminishing from the center to the periphery of the scaffold and the CV increasing from about 30% in the center to over 40% at the periphery. 

Previous studies showed that after 7–9 days in culture, PHHs seeded in these scaffolds maintain tight cell junctions and zonality (e.g., expression of specific transporters) and express albumin [40,42,43]. In our study, scaffolds (from experiments using HU8373 PHHs) were removed from the plate and fixed after 14 days of culture. Cells were stained with DAPI, anti-F-actin, anti-ZO-1, anti-CYP3A4, anti-BSEP, anti-Albumin, and anti-MRP2 antibodies (Figure 2D). We found that expression of tight junctions and other functional markers was maintained at 14 days of culture, albeit the uniformity of these imaging-based markers across scaffolds and experiments is difficult to ascertain because of the shape of the scaffolds (i.e., the depth of the channels allowed for visualization of only the apical-most layer of cells).

### 3.2. Comparison of the Basal Liver Function among Cell Types and MPS Platforms

**Figure 3:**
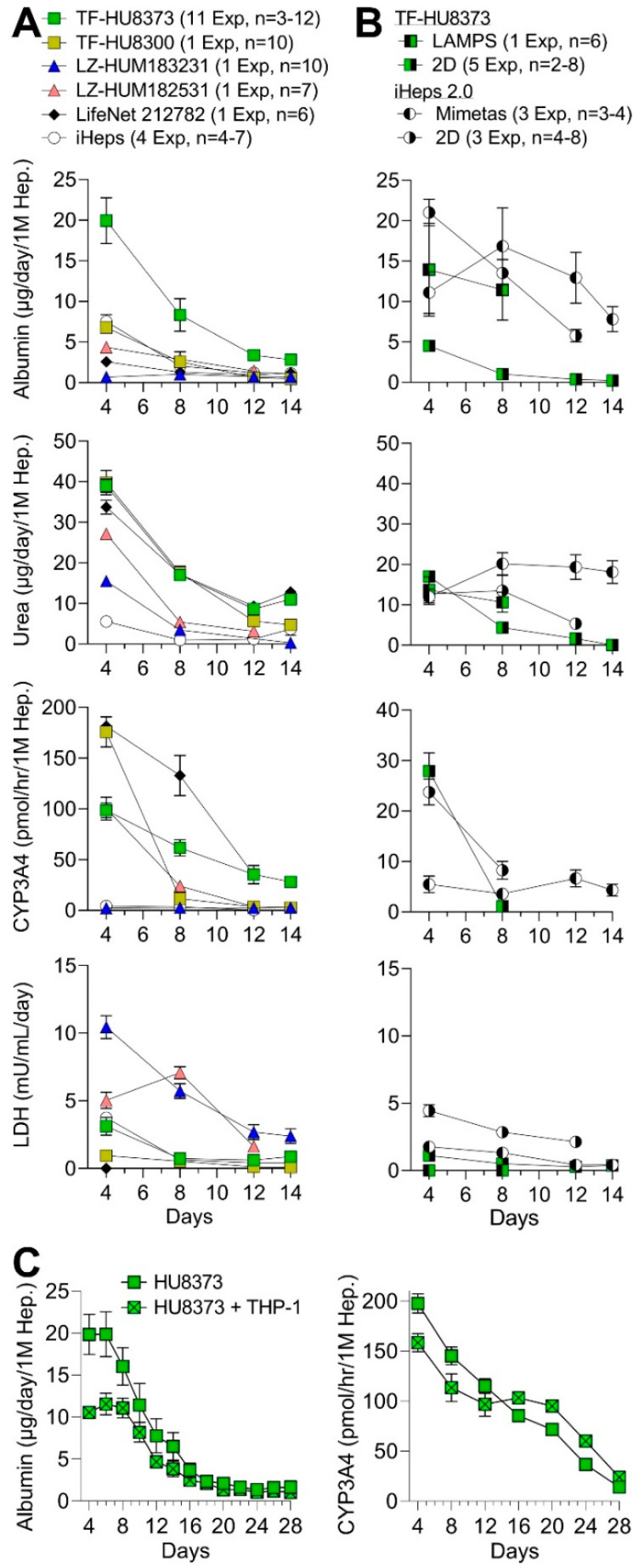
Comparison of basal function across different cell sources and models. (**A**) Albumin and urea secretion, CYP3A4 activity, and LDH leakage in the PhysioMimix LC12 under monoculture conditions across 5 different PHH donors and iPSC-derived hepatocytes (iHeps 2.0). (**B**) Albumin and urea secretion, CYP3A4 activity, and LDH leakage in 2D culture or other liver MPS models (LAMPS or OrganoPlate^®^ 2-lane 96) under monoculture conditions using either PHH donor HU8373 or iHeps. (**C**) Albumin secretion and CYP3A4 activity in the PhysioMimix LC12 under monoculture and co-culture conditions using PHH donor HU8373 over 28 days of culture. The number of experiments included in each condition of use with a range of individual chips in each experiment is indicated in the labels.

For the donor-to-donor comparisons, the basal function of five PHH donors and iPSC-derived hepatocytes was tested in monoculture conditions in the PhysioMimix LC12 over 14 days (Figure 3A). Albumin and urea secretion were used to estimate hepatocyte synthetic function, CYP3A4 activity to estimate metabolic capacity, and LDH leakage to estimate the stability of cell culture. When comparing all cell sources and donors, the liver functional markers were highest on day 4 and gradually decreased over 14 days of culture. PHHs overall had higher hepatic function, albeit wide variability was observed among donors, while iPSC-derived hepatocytes had the lowest function in this MPS. Of the PHH donors, TF-HU8373 exhibited the highest synthetic function and intermediate, but more sustained over time, activity of CYP3A4. These cells also had low basal LDH leakage. Therefore, this donor was selected for all further studies using PHHs. 

We next performed experiments using these PHHs in a LAMPS liver MPS model [27,51] and in a traditional 2D monolayer culture for up to 14 days (Figure 3B). Also included in the comparison were iHeps 2.0 iPSC-derived hepatocytes cultured in the OrganoPlate^®^ 2-lane 96 [33] and in a 2D monolayer over 14 days. The latter comparisons were included because we have shown that the OrganoPlate^®^ 2-lane 96 model is not suitable for experiments with PHHs but performs well with iHeps 2.0 [26]. When comparing PHH donor HU8373 cultured in the PhysioMimix LC12, 2D culture, or LAMPS, the liver synthetic function was similar across both liver MPS models and higher in the MPS models than in 2D cultures. iHeps 2.0 showed robust synthetic function in both OrganoPlate^®^ 2-lane 96 and 2D cultures, similar to that of PHH HU8373 in both LAMPS and PhysioMimix LC12. However, CYP3A4 activity of both PHH HU8373 in 2D, or iHeps 2.0 in other models was about a third of that for PHH HU8373 cultured in the PhysioMimix LC12 and diminished rapidly after 4 days in culture.

The ability of liver MPS to maintain cell functionality long-term is a highly desired feature for studies of sub-chronic exposures and pharmacokinetics of slowly metabolized drugs [42,52]. Most previous studies in LiverChip or PhysioMimix LC12 versions of this MPS have been performed for 7–14 days, with one study extending cultures to 19 days [41]. However, some liver models, such as the micropatterned co-culture of hepatocytes with fibroblasts [53], are functional for durations exceeding 30 days. To determine if PhysioMimix LC12 MPS maintains functionality beyond 2 weeks, we cultured HU8373 PHHs, with or without THP-1 cells, for up to 28 days (Figure 3C). Under these conditions, albumin production by hepatocytes gradually diminished after 8 days in culture and was very low after 14 days. CYP3A4 activity was preserved for up to 21 days but declined rapidly thereafter. 

**Figure 4:**
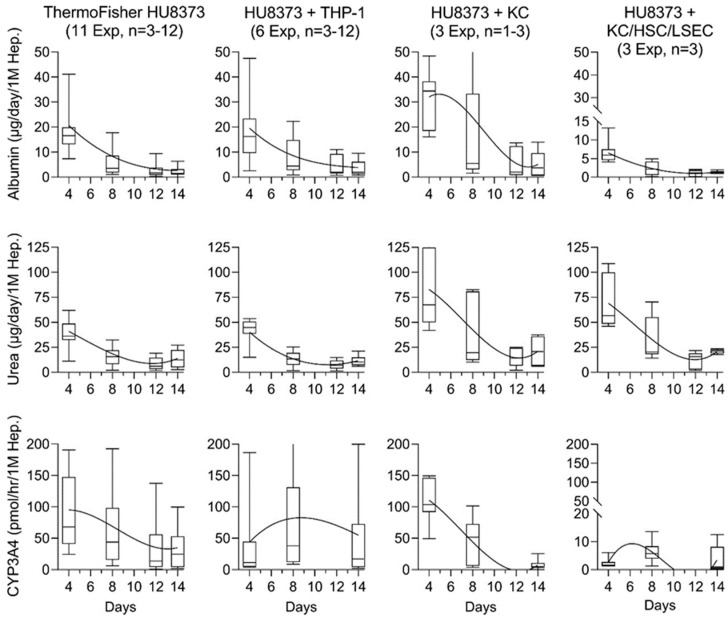
Inter-experimental variability of synthetic function and metabolic activity across replicate experiments and different conditions of use in the PhysioMimix LC-12. Time trends and variability in repeat experiments for albumin, urea, and CYP3A4 activity parameters in the PhysioMimix LC-12 across different conditions of use (PHH, PHH + THP-1, PHH + KC, or PHH + KC + HSC + LSEC). PHH donor HU8383 was used for all studies. Values are graphed as median (horizontal line), box (inter-quartile range), and whisker (10–90 percentile) plots by combining data from all studies for each PHH cell type combination. A best-fit power function is plotted to indicate power trends. The number of experiments included in each condition of use with a range of individual chips in each experiment is indicated in the labels.

Most previous studies in LiverChip or PhysioMimix LC12 versions of this MPS did not report inter-experimental variability in cell performance. A recent study that repeated experiments with one PHH donor five times reported albumin production on day 6 after cell seeding into the devices – they showed that after normalizing for cell seeding, intra-experimental reproducibility (expressed as coefficient of variability, CV) ranged from 2.5 to 20%, and was 22% across all replicate experiments [42]. Here, we provide a more comprehensive dataset (Figure 4) on the inter-experimental variability when using PhysioMimix LC12 MPS with the same donor (HU8373) and in different conditions of use (monoculture and co-culture with different types of NPCs). In monoculture conditions with HU8373 PHHs, we found the widest range of albumin, urea, and CYP3A4 on day 4 in culture; however, the CV was, on average, 20% or less (Supplemental Figure S1). Although the ranges tightened as the experiment continued, the CVs increased to 40% or more because the actual measured values decreased. In co-culture conditions of HU8373 PHHs with THP-1 cells, the trends in albumin and urea, as well as their average values, were similar to those in HU8373 PHHs cultured alone. However, two important differences were noted – the average CV across six experiments remained constant at ~20%, and CYP3A4 activity remained higher (albeit variable) for over 14 days. In experiments where HU8373 PHHs were co-cultured with primary human Kupffer cells, both albumin and urea were generally highest in the first week in culture; subsequently, they diminished gradually and varied between experiments. The activity of CYP3A4 was not sustained beyond one week in these co-cultures. Finally, in co-culture experiments of HU8373 PHHs with human Kupffer cells, HSCs, and LSECs, only urea production showed similar trends to other conditions; albumin and CYP3A4 levels were very low. The data for similar experiments with iHeps 2.0 in the PhysioMimix LC12 MPS are included in Supplemental Figure S2; the trends in variability in these experiments were similar to the experiments with PHH, albeit the levels of albumin, urea, and CYP3A4 were lower.

**Figure 5:**
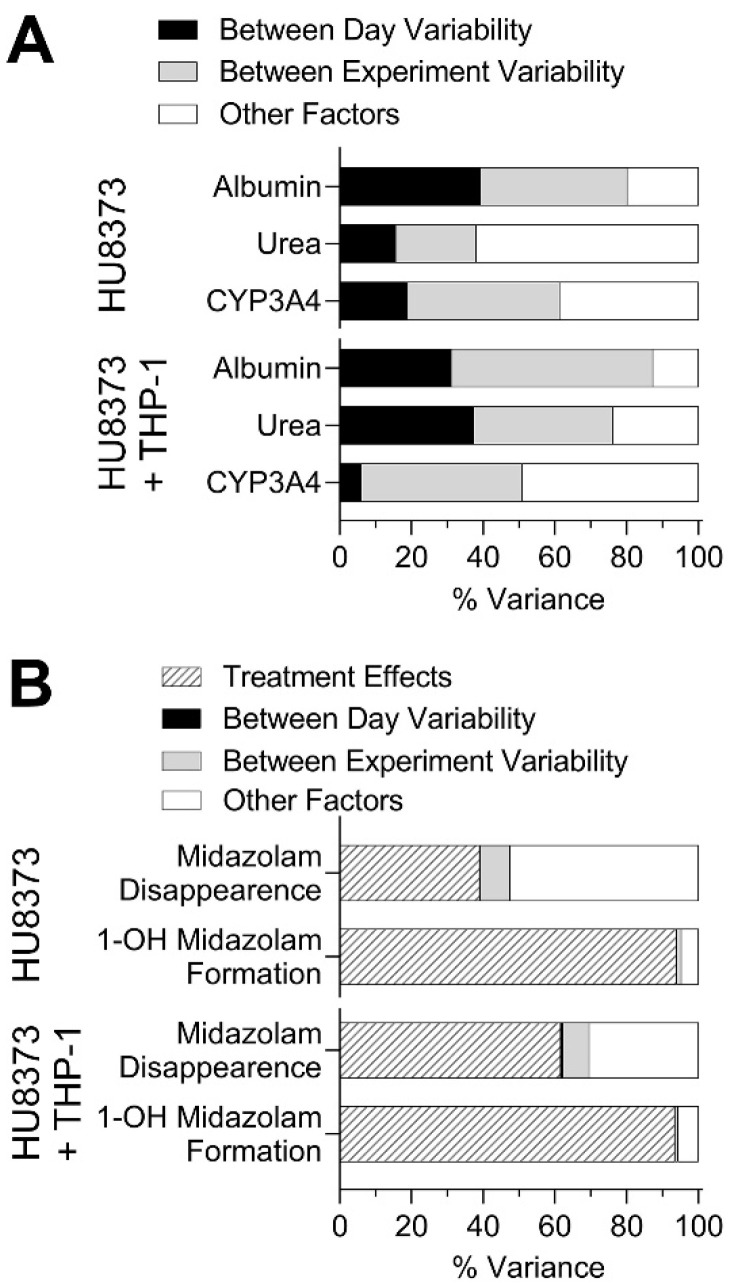
Contribution of different factors to overall variance in replicate experiments. The relative contribution of different factors (between day variability, between experiment variability, treatment effects, and other factors) to the overall variance of hepatic synthetic function and CYP3A4 activity (**A**) and midazolam metabolism (**B**) across replicate experiments. Sources of variability are indicated in the legends of each panel.

Using the replicate experiments in monoculture of HU8373 PHHs and co-culture of these PHHs with THP-1 cells, a power analysis was performed to determine the number of replicates that would be needed to observe significant differences in the basal phenotypes over time (Table 1). When using a standard assumption of requiring 80% power and a 5% significance level, we found that more than seven replicates would be needed to discern the time-related differences in basal liver function and CYP3A4 activity; fewest samples were needed to observe differences between early (days 3–5) and late (day 14) time points. Based on these analyses, it is also evident that repeated collection of the media from the same devices (paired analyses) would necessitate fewer replicates. This finding suggests that future experiments should attempt to run all controls and treatments in one experiment; however, the throughput of the system may present a challenge to such study designs. Applying a multi-factor analysis of variance, we also calculated the relative contribution of different factors to the overall variance in these experiments (Figure 5A). We found that “between day” variability of the same experiments and “between experiment” variability in completely independent runs of the same experiment were similar, regardless of the condition of use, albeit the variability in CYP3A4 activity between days of the same experiment in HU8373 + THP-1 experiments was the lowest.

### 3.3. Comparison of Drug and Chemical Metabolism among Cell Types and MPS Platforms

Most of the previous publications in LiverChip or PhysioMimix LC12 versions of this MPS have examined the utility of these models for studies of drug metabolism and pharmacokinetics of drugs. Because of the wide extent of the types of drugs used and endpoints examined in previous studies with monocultures of PHHs, we focused instead on several additional and largely unexplored questions–the reproducibility and robustness of drug metabolism studies across studies and co-culture combinations, the comparison of drug metabolism in the PhysioMimix LC12 MPS and other liver models, and the metabolism of environmental chemicals, such as pesticides, in a mixture setting. 

**Figure 6:**
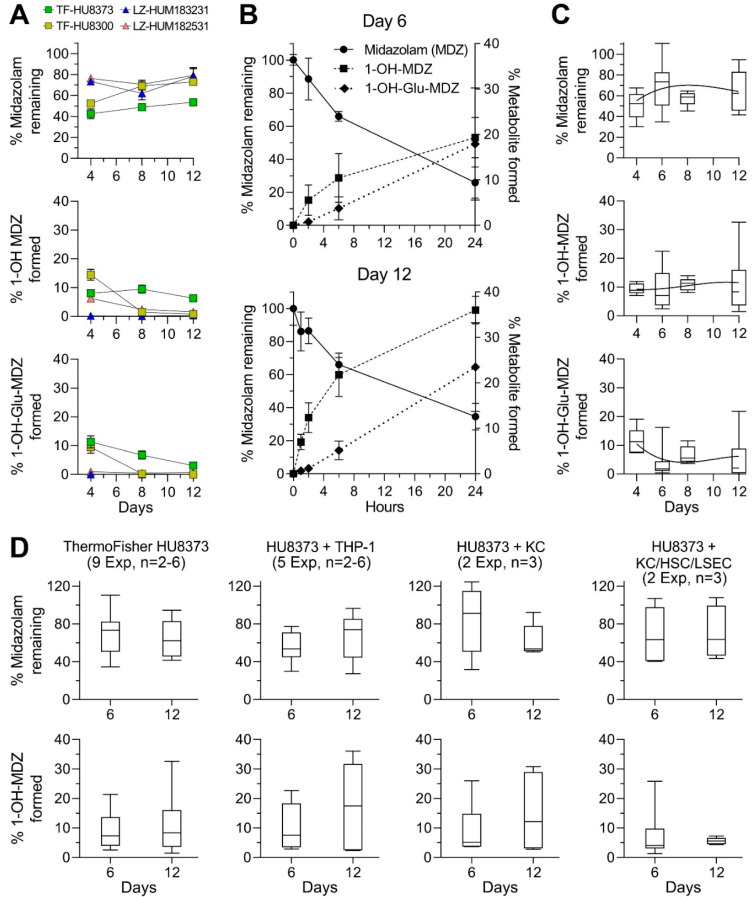
Metabolism of midazolam in the PhysioMimix LC-12. (**A**) Metabolism of midazolam (MDZ) is shown as % parent remaining and % metabolites formed (1′-OH MDZ and 1′OH MDZ Glucuronide) on Days 4, 8, and 12 at 24 h across 4 PHH donors. (**B**) Time course of midazolam metabolism over 24 h on Day 6 and Day 12 of a representative monoculture PHH (donor HU8373) experiment. (**C**) Intra-experimental variability of midazolam metabolism across time in monoculture PHH (donor HU8373) experiments. Values are graphed as box (interquartile range) and whisker plots (10–90 percentile) by combining data from all PHH monoculture studies at 24 h. (**D**) Intra-experimental variability of midazolam metabolism across time in PHH (donor HU8373) experiments across different conditions of use. Values are graphed as box (interquartile range) and whisker plots (10–90 percentile) by combining data for each cell combination at 24 h.

The metabolism of MDZ was compared among sources of PHHs in mono-culture PhysioMimix LC12 experiments over 12 days (Figure 6A). CYP3A4 activity was variable among these donor PHHs (Figure 3A), with donor HU8300 exhibiting the highest initial activity. However, this activity rapidly declined after 4 days. When MDZ metabolism was evaluated, we found that donor HU8373 exhibited the highest metabolism of the parent compound of all four donors; donor HU8300 yielded the greatest amount of 1′-OH-MDZ on day 4 in culture. Overall, donor HU8373 showed sustained metabolic function in both oxidation and glucuronidation of midazolam over 2 weeks, while other PHHs had largely lost meaningful metabolic capacity after one week. Then, we examined the kinetics of midazolam metabolism in HU8373 PHHs on days 6 and 12 in culture to determine whether the time course over 24 h was different (Figure 6B). We found that not only were the rates of metabolism similar over time but that the formation of 1′-OH-MDZ was even greater on day 12 than it was on day 6 in this experiment. Across repeat experiments (Figure 6C), we found that despite inter-experimental variability, the metabolic function was sustained. 

Next, midazolam metabolism was examined across different conditions of use (e.g., different cell combinations) in the PhysioMimix LC12 system. We found (Figure 6D) that midazolam metabolism remained consistent over time with similar levels of activity. However, the formation of 1′-OH-MDZ was the lowest when HU8373 was co-cultured together with KCs, HSCs, and LSECs. Previously published experiments utilizing iHeps in the OrganoPlate^®^ 2-lane 96 model system [26] also demonstrated sustained metabolism of midazolam to 1′-OH-MDZ at levels of formation similar to those reported here. 

The analysis of variability in these experiments confirmed that the variability between days was negligible, but variability between experiments was typically much greater (Figure 5B). Intra-experimental variability had a wide range in CVs (0.3 to 60%, Supplemental Figure S3). The power analysis (Table 2) indicated that studies examining the formation of 1′-OH-MDZ could yield informative results with as few as three replicates in paired analyses (i.e., sampling of the same devices over time). However, studies focusing on the disappearance of the parent molecule may require 4 to 11 replicates depending on the day of sampling and culture condition, with the PHH + THP-1 condition requiring the fewest replicates. 

Midazolam metabolism is a common biomarker of PHH functionality and has been reported for different PHH donors [54]. Rates of 1′-OH-MDZ formation have also been reported in other studies of LiverChip or PhysioMimix LC12 versions of this MPS [36], including comparisons among four PHH donors [38]. Although the experimental conditions across studies (e.g., donors, days in culture, and incubation length) varied and the formation of 1′-OH-MDZ is a supra-linear process (Figure 6B), we reason that the comparisons are informative (Table 3). It is well known that donor-to-donor variability is extensive, as was well documented by [54]. Another study [38] showed about a 3-fold difference among four donors. Our data for 6 and 12 days in culture can be most closely compared to [36] because in both cases, the formation of 1′-OH-MDZ was examined after 2 and 24 h of incubation and in similar culture time points (4 vs. 6 days). We found very similar rates although these experiments were performed years apart, with different PHH donors and different versions of this MPS (LiverChip vs. PhysioMimix LC12). These rates were comparable to general trends observed when comparing 2D cultures of HepaRG and sandwich cultures of primary human hepatocytes [54].

**Figure 7:**
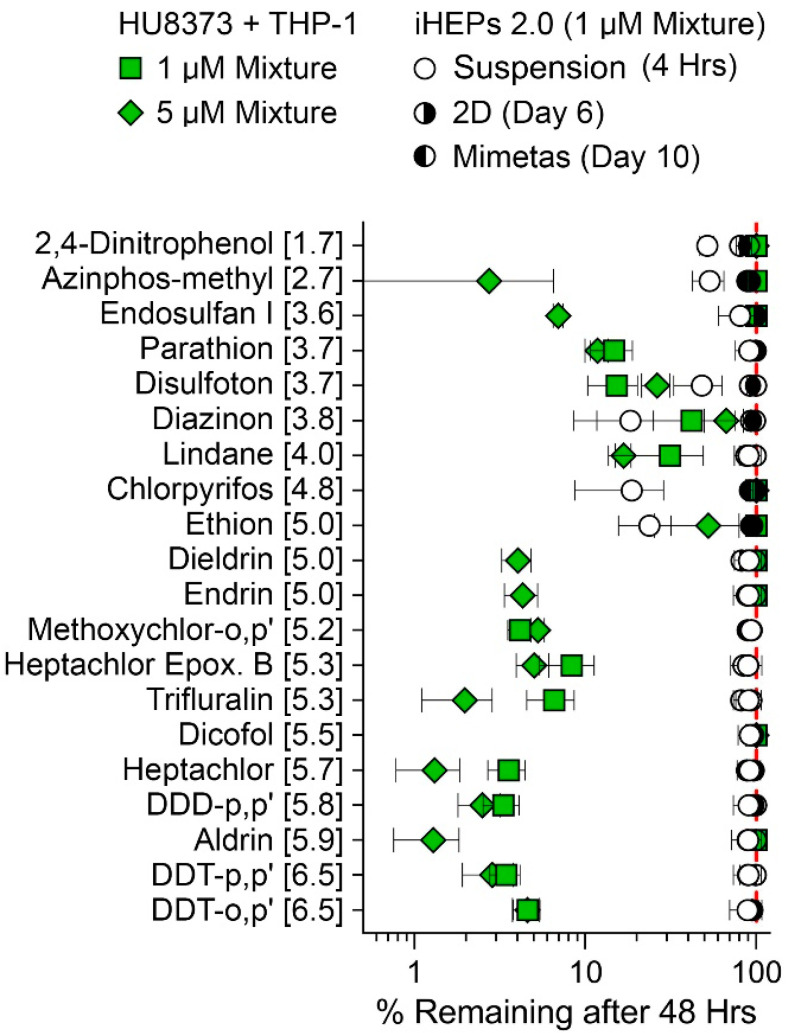
Metabolism of a mixture of 20 pesticides across different cell sources and models. Metabolism is shown as % parent compound remaining after 48 h in the PhysioMimix LC-12 using PHH (donor HU8373) + THP-1 with either 1 µM (green squares) or 5 µM (green diamonds) equimolar mixture and compared to the published results in [48] of experiments with iPSC-derived hepatocyte (iHeps 2.0) suspension, 2D culture, and OrganoPlate^®^ 2-lane 96 (abbreviated as “Mimetas”) experiments. Data shown are mean ± standard deviation (n = 3 in each condition).

We also examined the metabolism of a mixture of 20 pesticides from different chemical classes and of different hydrophobicity with the HU8373 PHH mono-culture condition in the PhysioMimix LC12 and compared this to the published results of experiments with iHeps suspensions, 2D cultures, and OrganoPlate^®^ 2-lane 96 experiments [44]. We examined metabolism in a mixture setting because previous studies using suspensions of PHHs showed that hepatocyte metabolic clearance was slower under mixture conditions when compared to the individual incubations with these pesticides [47]. To compare the timing of culture more closely to other studies in liver MPS, these experiments were conducted on day 6, and media was collected after 48 h for determination of the number of remaining parent compounds (Figure 1I). For almost all examined compounds (Figure 7), the disappearance of the parent molecules was far more evident in the experiments with HU8373 PHH in PhysioMimix LC12 when compared to iHeps 2.0 cultured in various models (suspension, 2D culture or OrganoPlate^®^ 2-lane 96).

### 3.4. A Study of Immune-Mediated Toxicity in PhysioMimix LC12

**Figure 8:**
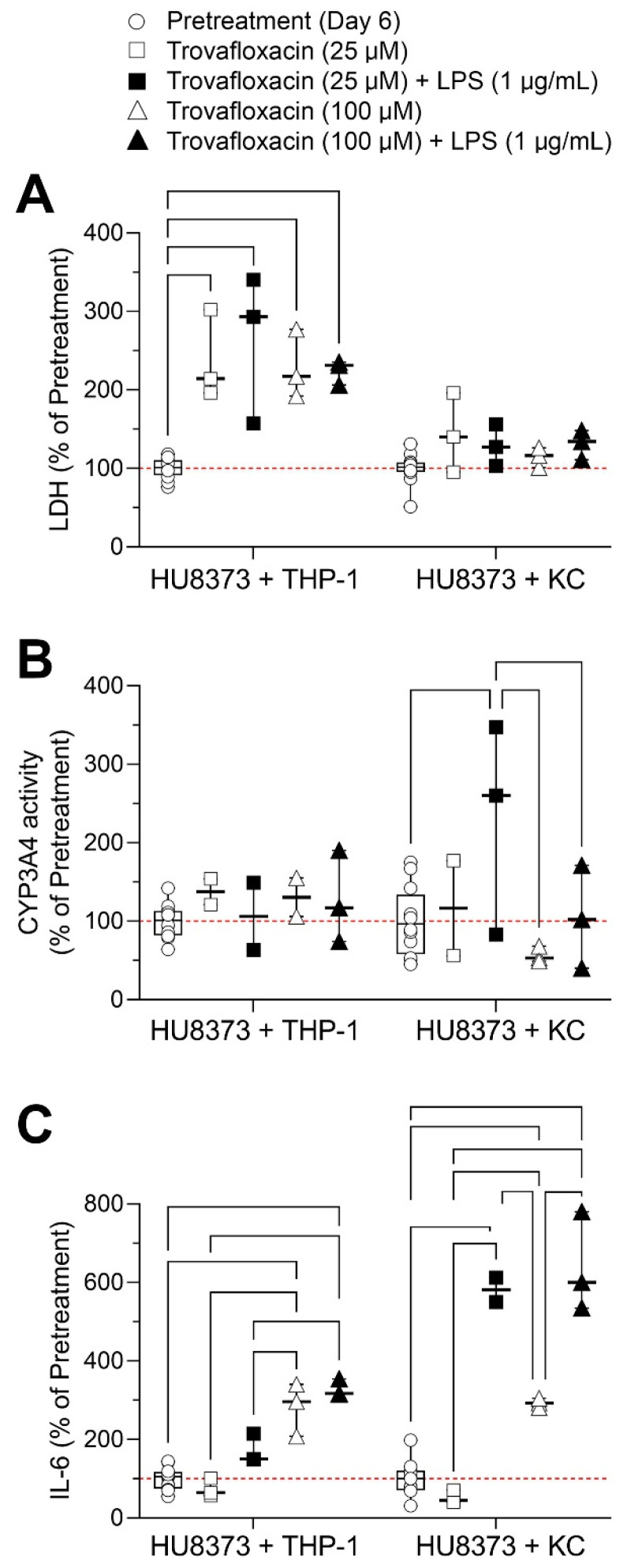
Immune-mediated toxicity in the PhysioMimix LC12. Changes in LDH, CYP3A4, and IL-6 after 48 h trovafloxacin exposure with and without LPS in the PhysioMimix LC12 seeded with PHHs (donor HU8373) and non-parenchymal cells (THP-1 cells or Kupffer cells). (**A**) LDH leakage, (**B**) CYP3A4 activity, and (**C**) IL-6 release. Endpoints were normalized to the mean of the pretreatment samples (day 6 of the experiment). Data are plotted as box (interquartile range) and whiskers (min-max) with all individual values shown (n = 2–12). The red dotted line shows 100% (mean of the pretreatment samples). Brackets indicate statistical significance between groups at *p* < 0.05 by two-way ANOVA with Tukey’s multiple comparisons test.

The last series of experiments was conducted to examine whether a co-culture of PHHs and NPCs in the PhysioMimix LC12 could model immune-mediated effects. Previous studies in the LiverChip [39] and PhysioMimix LC12 [41] provided evidence to suggest that this model has potential utility but that lab-to-lab and Kupffer cell donor variability exists [41]. We examined two conditions of use: HU8373 PHH with either THP-1 cells or Kupffer cells (Figure 8). Treatments (25 or 100 μM trovafloxacin) were added on day 8 with or without LPS (1 μg/mL) (Figure 1H) to replicate a well-established model of immune-mediated hepatotoxicity [55]. We examined LDH leakage as a marker of cytotoxicity, CYP3A4 activity, and IL-6 secretion into the media as a marker of pro-inflammatory response. Significant increases in LDH were observed in all treated groups as compared to pre-treatment levels only in PHH + THP-1 experiments (Figure 8A). Few effects on CYP3A4 were observed, albeit inter-experimental variability was high (Figure 8B). Increases in IL-6 were observed in both co-culture types with LPS alone, 100 μM trovafloxacin, or a combination of the two; however, a significant potentiating effect of LPS on trovafloxacin (at both concentrations) was observed only in the experiments with primary human Kupffer cells, not THP-1 cells (Figure 8C), which was consistent with the results of [56].

## 4. Discussion

A large number of liver MPS models have been developed in an attempt to create more physiological and translatable non-animal approaches that can be used in pre-clinical studies of drug metabolism and toxicology to improve benefit/risk assessment [29,30,52]. The primary decisive factors to model liver tissue have been recently described [31] and include (i) the creation of models that are multi-cellular in nature, allow for proper zonation, and can be used to study disruptions in bile acid homeostasis, (ii) evaluation of potential liver toxicity (including innate immune-mediated), and (iii) characterization of drug metabolism and pharmacokinetics, especially for slowly biotransformed compounds. Despite the diversity in the offerings of MPS and other more traditional and simpler in vitro liver models [29,57], a consensus on what models are useful, robust, reproducible, and fit for at least some regulatory decision making has yet to emerge. There is a clear shift from the use of liver MPS for hypothesis-driven research to their application in the laboratories of the potential end users, namely the pharmaceutical industry [14]. Therefore, building confidence in the wide adoption of these new models is needed; however, the approaches to how this could be accomplished vary. Some have taken a path to demonstrate their model’s utility via testing of a large number of liver-toxic compounds and establishing “performance” against human liver toxicity data [32,33], while others have taken a lab-to-lab comparison approach to focus on reproducibility, robustness, and determination of the potential challenges with model performance to inform future users on the most optimal study designs, conditions of use, and set overall expectations [27,41]. Indeed, both approaches are needed and address different considerations for the ultimate human translation and wide adoption of MPS.

The studies detailed herein are of the latter type; we aimed to independently evaluate a relatively mature liver MPS, the PhysioMimix LC12. This perfused scaffold-based model has over a decade-long track record of publications from both the developer lab [34,35,37,39,40,50] and various end users of this technology after it was commercialized [36,38,41,42]. While we relied on the manufacturer’s protocols, the conditions of use for the PhysioMimix LC12 were adjusted based on the considerations of the members of the TEX-VAL Tissue Chip Testing Consortium, a collaboration that brings together pharmaceutical and consumer products companies, the trade association of chemical manufacturers, and government agencies who collectively work to qualify a number of MPS [20]. The Consortium aims to not only conduct qualification experiments on a number of commercially available platforms, but also to create a transparent record of the process and make all data available by depositing it into a publicly accessible database [58]. 

In addition, the data presented here is directly responsive to the regulatory agencies’ calls for new MPS and other non-animal models [11,12,24,25]. Building scientific confidence in the regulatory use of new methods is an evolving field that is informed by authoritative bodies such as the 2023 US National Academies Report “Building Confidence in New Evidence Streams for Human Health Risk Assessment: Lessons Learned from Traditional Toxicity Tests” [59] and the 2005 OECD Guidance Document on the Validation and International Acceptance of New or Updated Test Methods for Hazard Assessment [60]. Thus, we interpret the findings of our study in the setting of the specific decision contexts and frame the qualification-relevant results around both PECO [relevant human population (P), exposure (E), comparator (C), and outcome (O)] criteria and critical elements of the qualification framework proposed by the National Academies [59]. With respect to the decision contexts, our studies aimed to characterize the reproducibility, robustness, and human translational relevance of the PhysioMimix LC12 model in the contexts of (i) varying conditions of use (i.e., cell types and culture duration), (ii) drug metabolism and pharmacokinetics, and (iii) immune-mediated toxicity. This MPS model’s PECO statement elements were P = specific types of PHHs, iHeps, and NPCs; E = drugs and chemicals dissolved in media or solvents; C = vehicle-control or pre-treatment conditions; and O = imaging, biochemical, and analytical chemistry readouts. We evaluated this model’s internal validity (systematic error due to study design or execution limitations), external validity (whether the results from tested MPS reflect the expected changes in humans), experimental variability, and biological (between donor) variability. Finally, the transparency of this qualification exercise was supported not only by the data presented herein and in the Supplemental Files but also by the full access to the protocols and raw data for this large compendium of studies that is freely accessible online (Supplemental Table S1).

Our study provides important information for establishing the internal validity of this liver MPS, i.e., determining the extent to which systematic error can influence the interpretation of the results. Previous studies have demonstrated that while drug binding to the materials of the PhysioMimix LC12 model is not likely to be of concern [42], media evaporation during open-system recirculation needs to be taken into consideration during kinetic modeling. A very important internal validity challenge is the seeding efficiency within each scaffold and the resulting differences between scaffolds in one plate or parallel plates. We found that consistency in seeding of the scaffolds is difficult to achieve, especially when co-cultures with multiple NPCs are used. Media movement-induced sheer stress and re-oxygenation of the media are critical design elements that distinguish this MPS from other models or static hepatocyte or spheroid cultures [34,61]; thus, maintaining even media flow through the scaffold is critical. Indeed, uncontrollable variability in cell density within and among scaffolds in the PhysioMimix LC12 model is a confounding factor that is a challenge of this type of MPS. It has been estimated that only about 50% of the cells originally seeded (~600,000/scaffold for PHHs) remain within the culture system after 2 days of perfusion [42]; however, a wide range (39–67%) was reported by that study. Because accurate cell number is needed for calculating kinetic parameters, it was suggested that “sentinel” scaffolds be reserved on each plate to evaluate seeding density via protein measurements [42], a suggestion that may be difficult to implement if there is substantial variability between scaffolds on the same plate, as reported here and by others [41]. Imaging of the scaffolds at the end of each experiment is used to visualize tissue morphology inside each scaffold to determine whether cells are retained. However, scaffolds are non-translucent, which precludes one from determining if a large proportion of the cells are located on top of the scaffold rather than inside the channels. Another observation that was made in many of our studies is that air bubbles become trapped under some scaffolds and may impede the stable circulation of the media through the system. Collectively, our finding of the high intra- and inter-experimental variability in basal liver function parameters, even with the same PHH donor cells, should be contemplated by the end users when considering study designs and throughput. A very high demand for the number of cells to be used and the limits on how many plates can be handled simultaneously by each PhysioMimix controller or can be realistically handled by one operator place this MPS (in its current configuration) into a low-throughput category of the available liver in vitro models.

For considerations of establishing external validity or the extent to which the results from this MPS model can be applied (generalized) to the intended population for drug development/safety, we provide liver physiological parameter data for comparisons with other human liver MPS models. Common benchmarks of the physiology of liver in vitro models include the level and sustainability in the production of albumin and urea and the activity of major metabolic enzymes [31]. Quantitative comparisons to other studies in this liver MPS and to the expected human levels show that this model could achieve basal physiological function at the lower range of the in vivo human parameters. While our study did not account for the cell loss and normalized to the nominal (i.e., seeded) numbers, we found that the levels in the first week of culture are comparable to those reported for this liver MPS by others [36,39,41,42]. It is worth noting that the amount of albumin and urea produced in the PhysioMimix LC12 was comparable to those obtained in either LAMPS (using the same donor PHH) or in OrganoPlate^®^ 2-lane 96 (seeded with iPSC-derived hepatocytes). This collectively indicates that PhysioMimix LC12 may not hold a unique advantage in terms of basal synthetic liver function and that maintaining basal function over time is also a challenge. 

Previous studies in the PhysioMimix LC12 that included time-course data for albumin and urea production suggested that their production is sustained for 1 or 2 weeks; however, we found that regardless of the donor or co-culture conditions, these biomarkers gradually diminished with time, declining most notably after 2 weeks in culture. This may be due to the differences in PHH donors, but this finding is informative to future users who should consider limiting studies in this model to 14 days. We report similar observations for CYP3A4 activity. However, we found that MDZ metabolism remained high over 14 days and was comparable to other published studies. Because almost all previous studies in the PhysioMimix LC12 focused on investigations of drug metabolism and clearance, our findings suggest that this model may be most suitable for studies of pharmacokinetics over days to weeks, a necessity for compounds with slow metabolism and low clearance that cannot be evaluated in traditional hepatocyte suspension cultures.

Our data show that high experimental variability is to be expected in this MPS. By quantifying variability over many replicate experiments, as well as comparing the variability in different conditions of use, we provide realistic estimates of the replicates that need to be considered for any future study design. While not unexpected because of the complexity of the model, the end users also need to determine what decision context they are pursuing when using the PhysioMimix LC12. For example, the variability in the physiological parameters was far greater than that observed when measuring drug metabolism. However, the typical “3 replicates” study designs may not be sufficient, even for the most reproducible endpoints, because of the difficulties in controlling cell seeding and other technical challenges that may arise during experiments that extend over 2–3 weeks. 

Our study also offers information about biological variability. It is well known that both the physiological and metabolic function of primary liver parenchymal cells from different donors varies greatly [54] and that the performance of various cell lines or iPSC-derived hepatocytes is also far from the liver in vivo [62]. Suggestions have been made that co-cultures of PHHs with NPCs may improve the performance of in vitro models or that the use of pooled PHHs from different donors may be a sensible strategy. However, the field is still searching for a solution, and various MPS may offer only partial benefits [63,64]. 

In this respect, we found that co-cultures with NPCs in the PhysioMimix LC12 offer only a limited benefit but further increase the complexity and cost of the experiments for the biological response spaces addressed in this study. Specifically, the inclusion of innate immune cells, such as macrophages [65,66], or other non-parenchymal cells, such as hepatic stellate cells [67], has been suggested as a pre-requisite to the utility of liver MPS as models for drug-induced liver injury or cholestasis. While it is without a doubt that co-cultures are necessary to replicate a complex whole liver cell–cell interaction, many MPS are still challenging with respect to model setup and equipment needs, even when hepatocyte monoculture is used. We found that while adding macrophages, either THP-1 or Kupffer cells, may yield some benefits and not erode overall performance, the addition of more complex combinations of NPCs requires additional optimization, and its benefits and reproducibility are yet to be demonstrated. One previous study showed LPS-mediated potentiation of trovafloxacin hepatotoxicity in PhysioMimix LC12, where PHHs were co-cultured with Kupffer cells [41], albeit the results across laboratories and donors were variable. Our study showed that depending on the phenotyping method, such potentiation could be observed in cytokine release in experiments with Kupffer cells but not THP-1 cells.

## 5. Conclusions

This study contributes critical new knowledge on the Physio-Mimix LC12, a microphysiological platform designed for toxicological studies of liver biology and the effects of drugs and other xenobiotics. The primary innovation of this study lies in the comprehensive approach to model evaluation and the use of both PHHs and human induced pluripotent stem cell (iPSC)-derived hepatocytes, with and without the addition of NPCs. By performing multi-faceted phenotyping of both synthetic function and metabolic capacity of liver cells in this model, as well as comparing this model’s performance to multi-well plates and other liver MPS, we provide uniquely informative data that allow evaluation of robustness, reproducibility, and potential utility of the Physio-Mimix LC12 for studies of hepatic function, drug metabolism, and immune-mediated hepatotoxicity.

Overall, we conclude that despite high inter-experimental variability in traditional liver synthetic function parameters, the PhysioMimix LC12 MPS exhibits sustained liver metabolism capacity for up to 2 weeks and may be most suitable for studies of drug and chemical metabolism and pharmacokinetics. This model was tested in a variety of conditions of use, both in this study and in other published reports. Although it may not be suitable for screening purposes, conducting targeted analyses of selected compounds with sufficient number of replicates should yield informative data for certain decision contexts in drug and chemical safety evaluations.

## Figures and Tables

**Table 1:** Sample size estimates for observing time-related differences in basic hepatic function in experiments with the PhysioMimix LC12 MPS seeded with PHHs (HU8373) with and without THP-1 cells. Shown are the number of replicates needed for detecting significant (*p* < 0.05 with 80% power) differences between time points for paired (resampling of the same chip over time) and unpaired (comparing different chips) study designs.

		Albumin		Urea		CYP3A4	
		Paired	Unpaired	Paired	Unpaired	Paired	Unpaired
PHH	3–5 vs. 14–17	8	16	26	>50	7	22
	7–8 vs. 14–17	18	>50	>50	>50	15	>50
	12–13 vs. 14–17	>50	>50	28	>50	33	>50
PHH + THP-1	3–5 vs. 14–17	9	20	6	18	>50	>50
	7–8 vs. 14–17	11	46	17	>50	21	>50
	12–13 vs. 14–17	28	>50	>50	>50	-	-

**Table 2:** Sample size estimates for observing time-related differences in midazolam metabolism (disappearance of the parent or formation of 1′-OH midazolam) in experiments with the PhysioMimix LC12 MPS seeded with PHHs (HU8373) with and without THP-1 cells. Shown are the number of replicates needed for detecting significant (*p* < 0.05 with 80% power) differences between time points for paired (resampling of the same chip over time) and unpaired (comparing different chips) study designs.

		Day 6		Day 12	
		Paired	Unpaired	Paired	Unpaired
PHH	Midazolam loss	11	18	10	14
	1′-OH Midazolam formation	3	4	3	4
PHH + THP-1	Midazolam loss	4	6	6	12
	1′-OH Midazolam formation	3	4	3	4

**Table 3:** Rate of formation of 1′-OH-midazolam (MDZ) in the experiments with human hepatocytes. Shown are the data and experimental designs for several publications and this study.

Data Source	Cell Types(s)	Model Type	Day(s) of Culture	Incubation Time (h)	1′-OH MDZ Formed (pmol/min/1 M Hep.)	
					Mean ± S.D.	Median [Range]
[54]	PHH (various donors)	Suspension	0	0.17	100 ± 106	63.7 [1.54–593]
[38]	PHH (various donors)	PhysioMimix LC-12	7	1	n/a	[8,9,10,11,12,13,14,15,16,17,18,19,20,21,22,23,24,25,26] *
[36]	PHH (various donors)	LiverChip	4	2	4.55 ± 2.27 *	n/a
This study	PHH (TF-HU8373)	PhysioMimix LC-12	6	2	2.72 ± 1.75	2.33 [0.337–5.56]
This study	PHH (TF-HU8373) + THP-1	PhysioMimix LC-12	6	2	2.54 ± 1.64	2.11 [0.561–5.86]
[36]	PHH (various donors)	LiverChip	4	24	0.216 ± 0.054 *	n/a
This study	PHH (TF-HU8373)	PhysioMimix LC-12	6	24	0.610 ± 0.403	0.561 [0.090–1.40]
This study	PHH (TF-HU8373) + THP-1	PhysioMimix LC-12	6	24	0.836 ± 0.556	0.997 [0.166–1.68]
This study	PHH (TF-HU8373)	PhysioMimix LC-12	12	24	0.753 ± 0.612	0.649 [0.032–1.91]
This study	PHH (TF-HU8373) + THP-1	PhysioMimix LC-12	12	24	0.971 ± 0.910	0.933 [0.135–1.90]
This study	PHH (TF-HU8373)	96-well plate	2	24	0.163 ± 0.064	0.189 [0.041–0.230]
This study	PHH (TF-HU8373) + THP-1	96-well plate	2	24	0.088 ± 0.077	0.061 [0.021–0.246]

Asterisks (*) denote the data that was estimated from the figures in the publications referenced in each row.

## Data Availability

The data presented in this study are openly available and can be found on the BioSystics-Analytics Platform (previously the MPS-Database). Links for each study are listed in Table S1.

## Supplementary Materials

The following supporting information can be downloaded at https://www.mdpi.com/article/10.3390/bioengineering10101195/s1. Figure S1: Intra-experimental variability (expressed as CV) of synthetic function and metabolic activity when using the PhysioMimix LC12 MPS with the same donor (HU8373) across different conditions of use, Figure S2: Inter- and intra- experimental variability of synthetic function and metabolic activity when using the PhysioMimix LC12 MPS with iHeps with and without NPCs, Figure S3: Intra-experimental variability (expressed as CV) of metabolic function when using the PhysioMimix LC12 MPS with the same donor (HU8373) across different conditions of use; Table S1: List of experiments included in each figure and the links to study protocols and data, Table S2: Cell types used in these studies, Table S3: Test chemicals and internal standards used in the study of mixtures of 20 pesticides.
