# Supplementary material for: Reproducibility and Robustness of a Liver Microphysiological System PhysioMimix LC12 under Varying Culture Conditions and Cell Type Combinations
Source: Bioengineering (Basel). 2023 Oct 14;10(10):1195. doi: 10.3390/bioengineering10101195 (PMC10603899; doi:10.3390/bioengineering10101195)

Supplemental Table S1. List of experiments included in each figure and the links to study protocols and data.

Figure	Experiments included
2	CN Bio: 3,5,6,8,10,13,14,23,27
3	CN Bio: 3,4,5,6,10,12,13,16,17,19,20,21,23,24,25,27,28,30 (also includes LAMPS),31
	Mimetas: 6,8,12
4	CN Bio: 4,10,16,19,20,21,23,24,25,27,30 (also includes LAMPS)
5	CN Bio: 4,10,16,19,20,21,23,24,25,27,30 (also includes LAMPS)
6	CN Bio: 3,4,10,16,19,21,23,24,25,27
7	CN Bio: 22
8	CN Bio: 28

Exp#	Experiment name	Database link
	**CNBio Experiments**	
3	Liver CNBIO LC12 Study 1: TEX-VAL-PK-2021-08-19-Liver_CNBIO LC12_Exp.3_PHH (LZ HUM183231) with Midazolam	https://mps.csb.pitt.edu/assays/assaystudy/593/
4	Liver CNBIO LC12 Study 2: TEX-VAL-PK-2021-09-12-Liver_CNBIO LC12_Exp.4_PHH (TF-HU8373) with Midazolam	https://mps.csb.pitt.edu/assays/assaystudy/594/
5	Liver CNBIO LC12 Study 3: TEX-VAL-PK-2021-09-19-Liver_CNBIO LC12_Exp.5_PHH (TF-HU8300) with Midazolam	https://mps.csb.pitt.edu/assays/assaystudy/679/
6	Liver CNBIO LC12 Study 4: TEX-VAL-PK-2021-09-30-Liver_CNBIO LC12_Exp.6_PHH (LZ HUM182531) with Midazolam	https://mps.csb.pitt.edu/assays/assaystudy/678/
8	Liver CNBIO LC12 Study 5: TEX-VAL-CC-2021-11-24-Liver_CNBIO LC12_Exp.8_PHH (TF-HU8373) ± THP-1 with LPS	https://mps.csb.pitt.edu/assays/assaystudy/701/
10	Liver CNBIO LC12 Study 7: TEX-VAL-PK-2021-11-29-Liver_CNBIO LC12_Exp.10 PHH (TF-HU8373) with Midazolam_2nd	https://mps.csb.pitt.edu/assays/assaystudy/728/
12	Liver CNBIO LC12 Study 9: TEX-VAL-PK-2022-01-16-Liver_CNBIO LC12_Exp.12_iHep with Midazolam_2nd	https://mps.csb.pitt.edu/assays/assaystudy/778/
13	Liver CNBIO LC12 Study 10: TEX-VAL-PK-2022-02-18-Liver_CNBIO LC12_Exp.13_iHep ± NPCs with Midazolam	https://mps.csb.pitt.edu/assays/assaystudy/894/
14	Liver CNBIO LC12 Study 11: TEX-VAL-CC-2022-02-19-CNBIO LC12_Exp.14_Exp.14 PHH + THP-1, Trovafloxacin ± LPS (2nd)	https://mps.csb.pitt.edu/assays/assaystudy/965/
16	Liver CNBIO LC12 Study 13: TEX-VAL-PK-2022-06-01-CNBIO LC12_Exp.16_PHH ± THP-1 with Midazolam	https://mps.csb.pitt.edu/assays/assaystudy/966/

17	Liver CNBIO LC12 Study 14: TEX-VAL-PK-2022-06-08-CNBIO LC12_Exp.17_iHep ± NPCs with Midazolam (2nd)	https://mps.csb.pitt.edu/assays/assaystudy/968/
19	Liver CNBIO LC12 Study 16: TEX-VAL-PK-2022-06-21-CNBIO LC12_Exp.19_PHH ± THP-1 with Midazolam (2nd)	https://mps.csb.pitt.edu/assays/assaystudy/969/
20	Liver CNBIO LC12 Study 17: TEX-VAL-PK-2022-07-19-CNBIO LC12_Exp.20_PHH ± primary NPCs, basal function (2nd)	https://mps.csb.pitt.edu/assays/assaystudy/970/
21	Liver CNBIO LC12 Study 18: TEX-VAL-PK-2022-07-19-CNBIO LC12_Exp.21_PHH ± THP-1 with Midazolam (3rd)	https://mps.csb.pitt.edu/assays/assaystudy/971/
22	Liver CNBIO LC12 Study 19: TEX-VAL-PK-2022-07-28-CNBIO LC12_Exp.22_PHH ± THP-1 with 20 pesticide mixture	https://mps.csb.pitt.edu/assays/assaystudy/972/
23	Liver CNBIO LC12 Study 20: TEX-VAL-PK-2022-08-13-CNBIO LC12_Exp.23_PHH ± primary NPCs, basal function (3rd)	https://mps.csb.pitt.edu/assays/assaystudy/973/
24	Liver CNBIO LC12 Study 21: TEX-VAL-PK-2022-09-13-CNBIO LC12_Exp.24_PHH ± THP-1 with Midazolam (4th), old vs. new plate	https://mps.csb.pitt.edu/assays/assaystudy/974/
25	Liver CNBIO LC12 Study 22: TEX-VAL-PK-2022-09-21-CNBIO LC12_Exp.25_PHH ± primary NPCs, basal function (4th)	https://mps.csb.pitt.edu/assays/assaystudy/975/
27	Liver CNBIO LC12 Study 24: TEX-VAL-PK-2022-11-02-CNBIO LC12_Exp.27_PHH ± THP-1 with Midazolam; 2 PHH donors	https://mps.csb.pitt.edu/assays/assaystudy/978/
28	Liver CNBIO LC12 Study 25: TEX-VAL-CC-2022-12-02-CNBIO LC12_Exp.28_PHH + THP-1, Trovafloxacin ± LPS (3rd)	https://mps.csb.pitt.edu/assays/assaystudy/979/
30	Liver CNBIO LC12 Study 27: TEX-VAL-CC-2023-01-24-CNBIO LC12_Exp.30_PHH ± THP-1, EaHy926, LX-2, vs. LAMPS	https://mps.csb.pitt.edu/assays/assaystudy/1043/
31	Liver CNBIO LC12 Study 28: TEX-VAL-PK-2023-04-14-CNBIO LC12_Exp.31_PHH ± THP-1 28-day study	https://mps.csb.pitt.edu/assays/assaystudy/1114/
**Mimetas Experiments**		
6	Liver Mimetas 2-lane Study 6: TEX-VAL-PK-2021-08-30-Liver_Mimetas 2-lane_Exp.6_iHep ± NPCs with Five Chemical Cocktail	https://mps.csb.pitt.edu/assays/assaystudy/680/
8	Liver Mimetas 2-lane Study 8: TEX-VAL-PK-2021-10-13-Liver_Mimetas 2-lane_Exp.8_iHep ± NPCs with Midazolam	https://mps.csb.pitt.edu/assays/assaystudy/681/
12	Liver Mimetas 2-lane Study 12: TEX-VAL-TOX-2022-02-11-Liver_Mimetas 2-lane_Exp.13_iHep and NPCs with Trovafloxacin ± LPS_2nd	https://mps.csb.pitt.edu/assays/assaystudy/759/
**LAMPS Experiments**		
30	Liver CNBIO LC12 Study 27: TEX-VAL-CC-2023-01-24-CNBIO LC12_Exp.30_PHH ± THP-1, EaHy926, LX-2, vs. LAMPS	https://mps.csb.pitt.edu/assays/assaystudy/1043/
**2D PHH Experiments**		

4	Liver CNBIO LC12 Study 2: TEX-VAL-PK-2021-09-12-Liver_CNBIO LC12_Exp.4_PHH (TF-HU8373) with Midazolam	https://mps.csb.pitt.edu/assays/assaystudy/594/
10	Liver CNBIO LC12 Study 7: TEX-VAL-PK-2021-11-29-Liver_CNBIO LC12_Exp.10 PHH (TF-HU8373) with Midazolam_2nd	https://mps.csb.pitt.edu/assays/assaystudy/728/
16	Liver CNBIO LC12 Study 13: TEX-VAL-PK-2022-06-01-CNBIO LC12_Exp.16_PHH ± THP-1 with Midazolam	https://mps.csb.pitt.edu/assays/assaystudy/966/
21	Liver CNBIO LC12 Study 18: TEX-VAL-PK-2022-07-19-CNBIO LC12_Exp.21_PHH ± THP-1 with Midazolam (3rd)	https://mps.csb.pitt.edu/assays/assaystudy/971/
27	Liver CNBIO LC12 Study 24: TEX-VAL-PK-2022-11-02-CNBIO LC12_Exp.27_PHH ± THP-1 with Midazolam; 2 PHH donors	https://mps.csb.pitt.edu/assays/assaystudy/978/
**2D iHep Experiments**		
12	Liver CNBIO LC12 Study 9: TEX-VAL-PK-2022-01-16-Liver_CNBIO LC12_Exp.12_iHep with Midazolam_2nd	https://mps.csb.pitt.edu/assays/assaystudy/778/
13	Liver CNBIO LC12 Study 10: TEX-VAL-PK-2022-02-18-Liver_CNBIO LC12_Exp.13_iHep ± NPCs with Midazolam	https://mps.csb.pitt.edu/assays/assaystudy/894/
17	Liver CNBIO LC12 Study 14: TEX-VAL-PK-2022-06-08-CNBIO LC12_Exp.17_iHep ± NPCs with Midazolam (2nd)	https://mps.csb.pitt.edu/assays/assaystudy/968/

Supplemental Table S2. Cell types used in these studies.

Vendor	Cell type	Lot Number	Donor	Race	Age (yrs)	BMI	Tobacco History	Alcohol History	Drug History	Cause of Death	Other Info
Thermo Fisher	Primary Human Hepatocytes	HU8300	Male	Caucasian	31	21	Yes	Yes	Yes	n/a	
Thermo Fisher	Primary Human Hepatocytes	HU8373	Female	Caucasian	26	18.6	Yes	Yes	Yes	n/a	
Lonza	Primary Human Hepatocytes	HUM182531	Female	Caucasian	51	25.7	No	Social	No	n/a	
Lonza	Primary Human Hepatocytes	HUM183231	Female	Caucasian	50	28.8	No	Social	No	n/a	
LifeNet	Primary Human Hepatocytes	2122782-01	Female	Caucasian	31	27.8	No	No	No	CVA/ Stroke	
Cell Systems	Primary human liver sinusoidal endothelial cells	566.01.01. 01.1T	Female	Caucasian	27	24.9	n/a	n/a	n/a	n/a	no diabetes; no chemo
LifeNet	Primary human Kupffer cells	2118082	Female	Caucasian	58	27.1	Yes	no	no	Anoxia	
LifeNet	Primary human stellate cells	2118082	Female	Caucasian	58	27.1	Yes	no	no	Anoxia	
FujiFilm	iCell hepatocytes 2.0	103664, 104926	Female	Caucasian	<18	n/a	n/a	n/a	n/a	n/a	Healthy; fibroblast tissue source
ATCC	HMEC-1	n/a	Male	n/a	<1	n/a	n/a	n/a	n/a	n/a	Endothelial-like immortalized cell line originally isolated from the endothelium of the foreskin
ATCC	THP-1	70047549	Male	n/a	1	n/a	n/a	n/a	n/a	n/a	Monocyte isolated from peripheral blood from an acute monocytic leukemia patient

Supplemental Table S3. Test chemicals and internal standards used in the study of mixtures of 20 pesticides.

Chemical	Vendor	Purity	Catalog No.
Test chemicals			
Aldrin	Chem Service	97.9%	N-11049
DDD-p,p'	Sigma-Aldrich	≥98%	35486
DDT-o,p'	Chem Service	99.5%	N-12708
DDT-p,p'	Sigma-Aldrich	≥98%	31041
Dicofol	Sigma-Aldrich	≥98%	36677
Dieldrin	Sigma-Aldrich	≥95%	33491
Endosulfan I	Sigma-Aldrich	≥98%	32015
Endrin	Sigma-Aldrich	≥98%	32014
Heptachlor epoxide B	Chem Service	99.5%	N-12148
Heptachlor	Chem Service	98.6%	N-12147
Lindane	Sigma-Aldrich	≥96.5%	233390
Methoxychlor-o,p'	Sigma-Aldrich	≥98%	36161
Parathion	Chem Service	98.4%	N-12819
Trifluralin	Sigma-Aldrich	≥98%	32061
2,4-Dinitrophenol	Sigma-Aldrich	≥98%	34334
Azinphos-methyl	Sigma-Aldrich	≥95%	45333
Chlorpyrifos	Sigma-Aldrich	≥98%	45395
Diazinon	Sigma-Aldrich	≥98%	45428
Disulfoton	Sigma-Aldrich	≥98%	45460
Ethion	Sigma-Aldrich	≥95%	45477
Internal Standards			
Atrazine	Sigma-Aldrich	≥ 98%	45330
Benzo[a]anthracene	Sigma-Aldrich	≥ 98.5%	B2209
Terbutryn	Sigma-Aldrich	≥ 98%	45677
Mifepristone	Selleck Chem	>99%	S2606
Troglitazone	Sigma-Aldrich	≥ 98%	T2573

Supplemental Figure S1. Intra-experimental variability (expressed as CV) of synthetic function and metabolic activity when using the PhysioMimix^{TM} LC12 MPS with the same donor (HU8373) across different conditions of use.

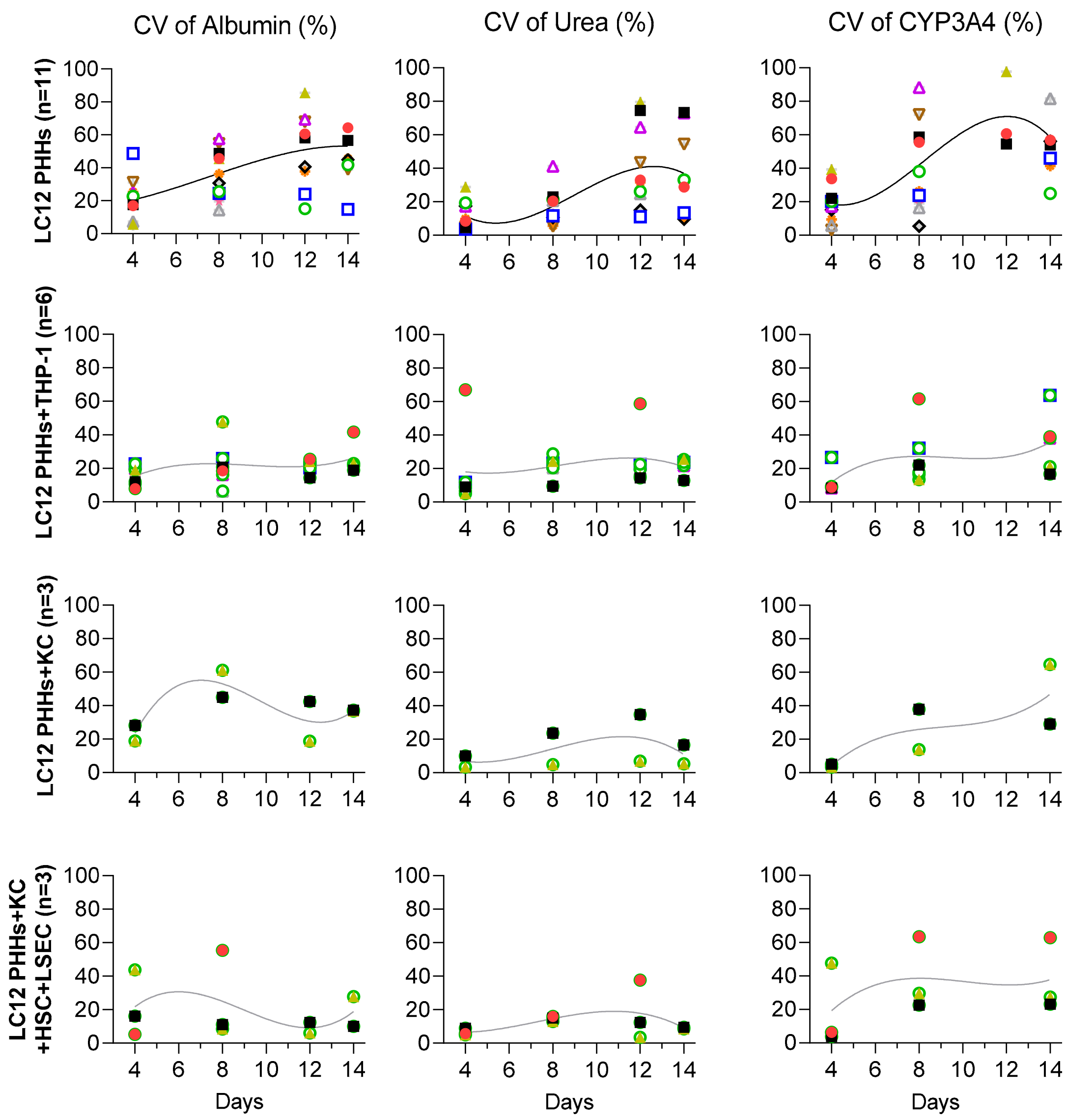

Supplemental Figure S2. Inter- and intra- experimental variability of synthetic function and metabolic activity when using the PhysioMimix^{TM} LC12 MPS with iHeps with and without NPCs.

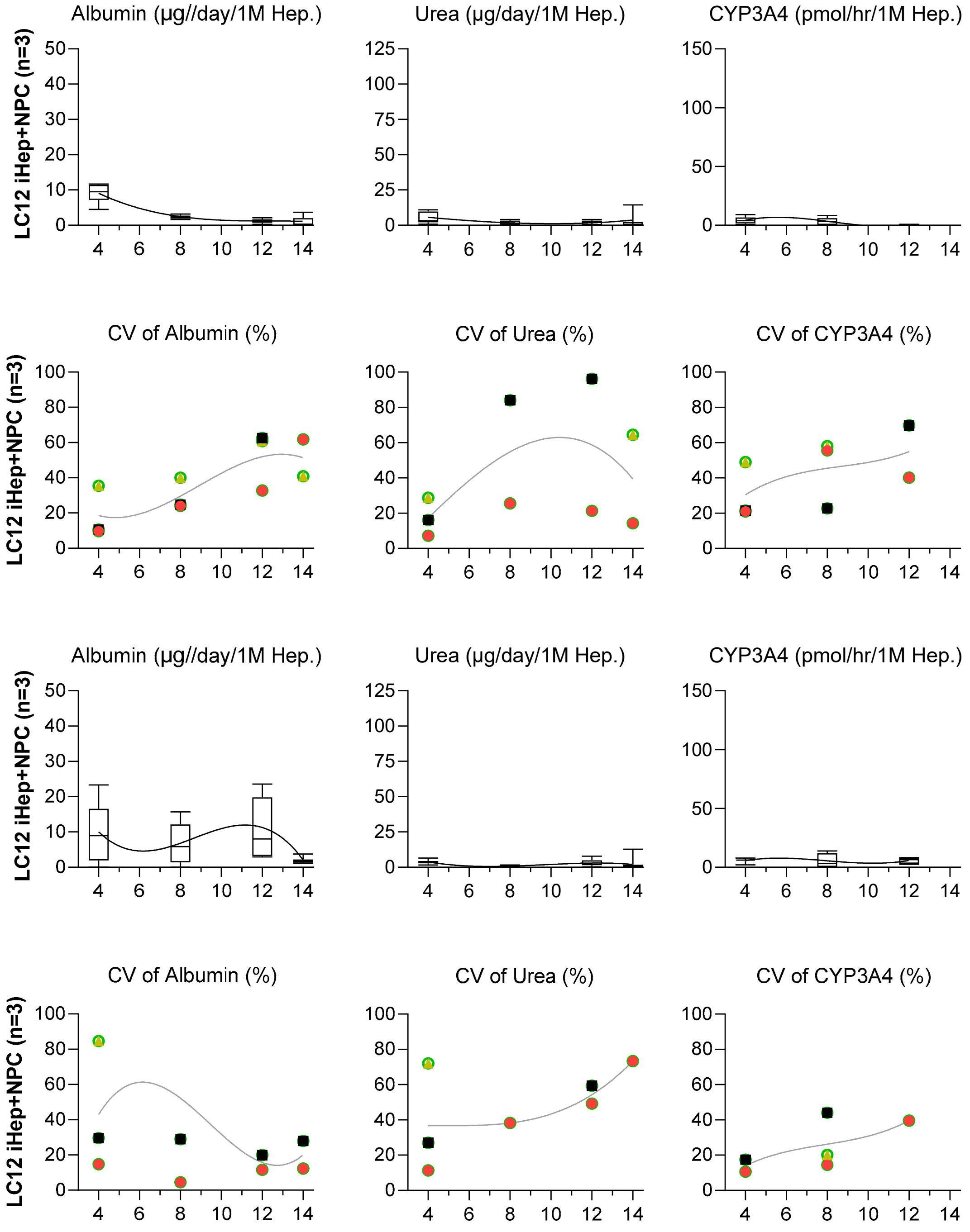

Supplemental Figure S3. Intra-experimental variability (expressed as CV) of metabolic function when using the PhysioMimix™ LC12 MPS with the same donor (HU8373) across different conditions of use.

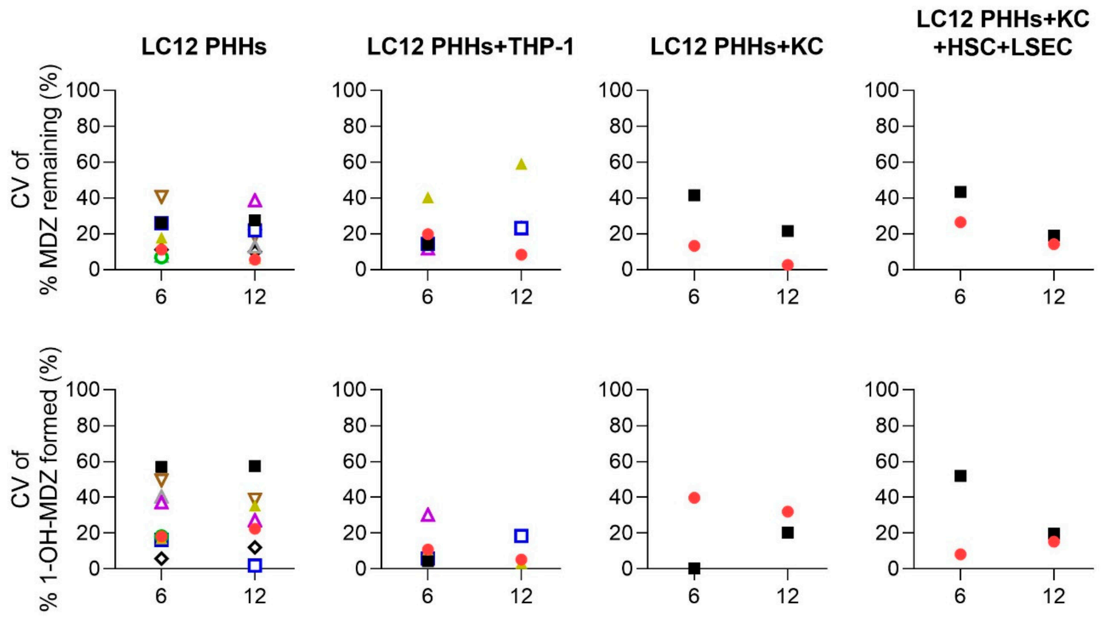

Supplement: Supplementary file 1 [file bioengineering-10-01195-s001.zip › bioengineering-2609352-supplementary.pdf]
